# Development and validation of an MRI spatiotemporal interaction model for early noninvasive prediction of neoadjuvant chemotherapy response in breast cancer: a multicentre study

**DOI:** 10.1016/j.eclinm.2025.103298

**Published:** 2025-06-12

**Authors:** Wenjie Tang, Chen Jin, Qingcong Kong, Chunling Liu, Siyi Chen, Shishen Ding, Bihua Liu, Zaihui Feng, Ying Li, Yi Dai, Lei Zhang, Yongxin Chen, Xiaorui Han, Shuang Liu, Dandan Chen, Zijin Weng, Weifeng Liu, Xinhua Wei, Xinqing Jiang, Qianwei Zhou, Ning Mao, Yuan Guo

**Affiliations:** a Department of Radiology, Guangzhou First People’s Hospital, South China University of Technology, Guangzhou, 510180, China; b College of Computer Science and Technology, Zhejiang University of Technology, Hangzhou, 310023, China; c Department of Radiology, The Third Affiliated Hospital, Sun Yat-Sen University, Guangzhou, 510630, China; d Department of Radiology, Guangdong Provincial People’ Hospital (Guangdong Academy of Medical Sciences), Southern Medical University, 510080, China; e Department of Radiology, Liuzhou People’s Hospital Affiliated to Guangxi Medical University, Liuzhou, 545006, China; f Department of Radiology, The Tenth Affiliated Hospital of Southern Medical University (Gongguan People’s Hospital), China; g Department of Radiology, The Third People’s Hospital of Honghe Hani and Yi Autonomous Prefecture, Honghe, 661000, China; h Department of Medical Imaging, Peking University Shenzhen Hospital, Shenzhen, Guangdong, 518036, China; i Department of Diagnostic Radiology and Nuclear Medicine, University of Maryland School of Medicine, MD, 21201, USA; j Department of Pathology, The Third Affiliated Hospital, Sun Yat-Sen University, Guangzhou, 510630, China; k Department of Radiology, Yantai Yuhuangding Hospital, Qingdao University, Yantai, Shandong, 264000, China

**Keywords:** Deep learning, Early prediction, Breast cancer, Neoadjuvant chemotherapy, Pathological complete response, Longitudinal MRI

## Abstract

**Background:**

The accurate and early evaluation of response to neoadjuvant chemotherapy (NAC) in breast cancer is crucial for optimizing treatment strategies and minimizing unnecessary interventions. While deep learning (DL)-based approaches have shown promise in medical imaging analysis, existing models often fail to comprehensively integrate spatial and temporal tumor dynamics. This study aims to develop and validate a spatiotemporal interaction (STI) model based on longitudinal MRI data to predict pathological complete response (pCR) to NAC in breast cancer patients.

**Methods:**

This study included retrospective and prospective datasets from five medical centers in China, collected from June 2018 to December 2024. These datasets were assigned to the primary cohort (including training and internal validation sets), external validation cohorts, and a prospective validation cohort. DCE-MRI scans from both pre-NAC (T0) and early-NAC (T1) stages were collected for each patient, along with surgical pathology results. A Siamese network-based STI model was developed, integrating spatial features from tumor segmentation with temporal dependencies using a transformer-based multi-head attention mechanism. This model was designed to simultaneously capture spatial heterogeneity and temporal dynamics, enabling accurate prediction of NAC response. The STI model’s performance was evaluated using the area under the ROC curve (AUC) and Precision-Recall curve (AP), accuracy, sensitivity, and specificity. Additionally, the I-SPY1 and I-SPY2 datasets were used for Kaplan–Meier survival analysis and to explore the biological basis of the STI model, respectively. The prospective cohort was registered with Chinese Clinical Trial Registration Centre (ChiCTR2500102170).

**Findings:**

A total of 1044 patients were included in this study, with the pCR rate ranging from 23.8% to 35.9%. The STI model demonstrated good performance in early prediction of NAC response in breast cancer. In the external validation cohorts, the AUC values were 0.923 (95% CI: 0.859–0.987), 0.892 (95% CI: 0.821–0.963), and 0.913 (95% CI: 0.835–0.991), all outperforming the single-timepoint T0 or T1 models, as well as models with spatial information added (all p < 0.05, Delong test). Additionally, the STI model significantly outperformed the clinical model (p < 0.05, Delong test) and radiologists’ predictions. In the prospective validation cohort, the STI model identified 90.2% (37/41) of non-pCR and 82.6% (19/23) of pCR patients, reducing misclassification rates by 58.7% and 63.3% compared to radiologists. This indicates that these patients might benefit from treatment adjustment or continued therapy in the early NAC stage. Survival analysis showed a significant correlation between the STI model and both recurrence-free survival (RFS) and overall survival (OS) in breast cancer patients. Further investigation revealed that favorable NAC responses predicted by the STI model were closely linked to upregulated immune-related genes and enhanced immune cell infiltration.

**Interpretation:**

Our study established a novel noninvasive STI model that integrates the spatiotemporal evolution of MRI before and during NAC to achieve early and accurate pCR prediction, offering potential guidance for personalized treatment.

**Funding:**

This study was supported by the 10.13039/501100001809National Natural Science Foundation of China (82302314, 62271448, 82171920, 81901711), 10.13039/501100021171Basic and Applied Basic Research Foundation of Guangdong Province (2022A1515110792, 2023A1515220097, 2024A1515010653), Medical Scientific Research Foundation of Guangdong Province (A2023073, A2024116), Science and Technology Projects in Guangzhou (2023A04J1275, 2024A03J1030, 2025A03J4163, 2025A03J4162); Guangzhou First People’s Hospital Frontier Medical Technology Project (QY-C04).

**Research in context**

**Evidence before this study**

We searched for the terms (“deep learning” OR “radiomics” OR “artificial intelligence”) AND (“breast cancer” OR “breast tumor”) AND (“neoadjuvant chemotherapy”) AND (“pathological complete response”) AND (“MRI”) without date or language restrictions on PubMed. We found that previous studies primarily relied on pre-treatment MRI images to predict the efficacy of neoadjuvant treatment for breast cancer. Between January 2021 and December 2024, only five studies on AI-based longitudinal MRI for predicting neoadjuvant treatment outcomes were published. However, these studies did not integrate the interactive and dynamic spatial and temporal characteristics of tumors during treatment and lacked a biological interpretability analysis of the models. This limits both the accuracy of tumor response predictions and the potential for clinical application. Therefore, there is a need to develop a non-invasive deep learning model that simultaneously captures the spatial heterogeneity and temporal dynamics of tumors, enabling early, precise, and personalized treatment predictions, and ultimately supporting individualized treatment decisions for breast cancer patients.

**Added value of this study**

In this multicenter retrospective and prospective study, we developed and validated a Spatiotemporal Interaction (STI) model that utilizes longitudinal MRI data to simultaneously capture the spatial heterogeneity and temporal dynamics of tumors for early prediction of neoadjuvant chemotherapy (NAC) response in breast cancer. By integrating both spatial and temporal features, our model provides a more comprehensive understanding of tumor behavior during treatment, significantly improving prediction accuracy compared to models based solely on a single time point or spatial information. Furthermore, the STI model outperformed clinical models and radiologists’ subjective assessments across multiple cohorts, and the biological mechanisms underlying the model were explored, providing biological interpretability for its predictions.

**Implications of all the available evidence**

Our study demonstrates that the STI model, by effectively integrating spatial and temporal features into a Siamese deep learning framework based on a transformer-based multi-head attention mechanism, can accurately predict early responses to NAC in breast cancer patients. The model also exhibits excellent generalization ability across multiple cohorts. Additionally, biological interpretability analysis of the STI model reveals that its predictions are strongly associated with immune activation patterns, including increased T-cell infiltration and enriched immune signaling pathways, further enhancing its potential for clinical application.

## Introduction

Breast cancer has become the most prevalent malignancy among women worldwide and remains the leading cause of cancer-related deaths in this group worldwide.^1^ Neoadjuvant chemotherapy (NAC) is a standard treatment for breast cancer, widely used for downstaging tumors and increasing breast conservation rates.^2^^,^^3^ Patients typically undergo breast surgery after six or eight full cycles of NAC, with efficacy assessed by postoperative pathology. Studies have shown that patients who achieve pathological complete response (pCR) have significantly better long-term clinical outcomes compared to those who do not achieve pCR.^4^^,^^5^ However, the response of breast tumors to NAC varies greatly among patients, with the overall rate of achieving pCR after NAC ranging from only 19% to 30%.^6^, ^7^, ^8^ Therefore, early prediction of NAC response in breast cancer can help non-pCR patients adjust their treatment plans in a timely manner and spare pCR patients from unnecessary breast surgery, thus improving personalized clinical management for breast cancer patients.^9^^,^^10^

The European Society of Breast Imaging and the National Comprehensive Cancer Network (NCCN) guidelines recommend breast MRI for assessing response to NAC.^11^^,^^12^ Dynamic contrast-enhanced MRI (DCE-MRI), which forms the basis of breast MRI, reflects hemodynamic and morphological changes following tumor treatment by capturing contrast agent uptake.^13^ The ACRIN 6657/I-spy trial demonstrated that volumetric measurement of tumor size had a great advantage in predicting tumor response early in treatment.^14^ Currently, clinical monitoring of NAC response relies on the Response Evaluation Criteria in Solid Tumors (RECIST) 1.1, which evaluates the percentage change in the maximum tumor diameter.^15^ However, due to the highly heterogeneous and complex nature of breast cancer, this diameter-based MRI measurement primarily reflects morphological changes and has limited capacity for early prediction of NAC response.^13^

The integration of artificial intelligence (AI) with tumor imaging provides a more objective and precise method for reflecting tumor heterogeneity, expanding traditional imaging techniques and has great potential for noninvasive assessment of tumor treatment efficacy.^16^^,^^17^ Tumor heterogeneity is a key factor influencing cancer treatment, particularly at the histological and genetic levels.^18^^,^^19^ However, measuring spatial heterogeneity at the macroscopic level has always been challenging. To more accurately measure this heterogeneity, “habitat imaging” quantifies spatial heterogeneity by dividing the tumor into sub-regions (habitats) with similar biological characteristics.^20^ Studies have shown significant differences in perfusion and metabolism across tumor regions, which are closely related to treatment response.^21^^,^^22^ For example, Wu et al.^23^ used perfusion MRI to identify low-perfusion areas, which are associated with poorer prognosis. Kim et al.^24^ applied a computer-aided diagnosis (CAD) system to analyze preoperative MRI scans, finding a strong correlation between CAD-extracted kinetic features and long-term prognosis. Shi et al.^25^ combined MRI radiomics with tumor heterogeneity indices to develop a model for predicting NAC response. Unlike previous studies, which primarily focused on perfusion-related heterogeneity, Shi et al. utilized both C-radiomics scores and the intratumoral heterogeneity (ITH) index, extracted from pre-treatment MRI scans, to better capture tumor complexity. By quantitatively analyzing spatial heterogeneity, habitat imaging not only reveals the complex internal structure of tumors but also provides a new perspective for early prediction of NAC efficacy.

However, the aforementioned studies primarily rely on monophasic medical images for prediction, overlooking the temporal evolution of tumors during treatment. Longitudinal MRI has obvious advantages in monitoring treatment efficacy, and can effectively capture the dynamic changes occurring during the treatment process and comprehensively reflect the heterogeneity of tumors at different time points. Some studies^26^, ^27^, ^28^ have utilized radiomics to extract differentiating quantitative features from breast MRI and employed longitudinal MRI to calculate changes (e.g., delta features) that reflect tumor heterogeneity during treatment. These studies have demonstrated that multi-timepoint imaging data significantly improve prediction accuracy compared to single-timepoint data. However, radiomics techniques rely on manually crafted features, which inherently limits their ability to capture the complex and deeply embedded patterns present in imaging data. This reliance on predefined features not only restricts the flexibility of radiomics but also hampers its potential to fully leverage the rich information contained in medical images. Recently, several other studies^29^, ^30^, ^31^ have successfully applied longitudinal medical imaging-based deep learning (DL) models to predict treatment efficacy and prognosis in breast cancer patients. These studies highlight the capability of DL to automatically extract high-dimensional, deep features from raw imaging data, integrate temporal information from pre- and post-treatment images, and capture intricate patterns and subtle changes in tumor morphology and heterogeneity. However, existing longitudinal DL models often focus on simple fusion strategies (e.g., image subtraction, delta features, or early/late fusion), which may be insufficient to model complex temporal interactions.

Therefore, our proposed spatiotemporal interaction (STI) model builds upon these foundations by introducing an explicit interaction mechanism—leveraging multi-head attention modules—to capture dynamic feature interactions across multiple time points. This design enables the model to more effectively learn spatiotemporal dependencies, thereby enhancing its predictive performance. We believe that this model will contribute to more accurate early treatment response assessments, facilitate clinical decision-making, optimize treatment plans, and support personalized treatment strategies for breast cancer patients (Supplementary Fig. S1).

## Methods

### Ethics

This study has been approved by the Ethics Committee of the Guangzhou First People’s Hospital (approval number for retrospective and prospective studies: S-2023-083-01). Four other hospitals, including Yantai Yuhuangding Hospital, Liuzhou People’s Hospital, Dongguan People’s Hospital, and Honghe Prefecture Third People’s Hospital, have accepted the decision of the Ethics Committee of the Guangzhou First People’s Hospital. Informed consent was waived for the retrospective study, and all patients from the prospective study provided written informed consent (Chinese Clinical Trial Registration Centre, ChiCTR2500102170). All public datasets were fully de-identified and publicly available, eliminating the need for additional ethical approvals.

### Patients and study design

**Fig. 1:**
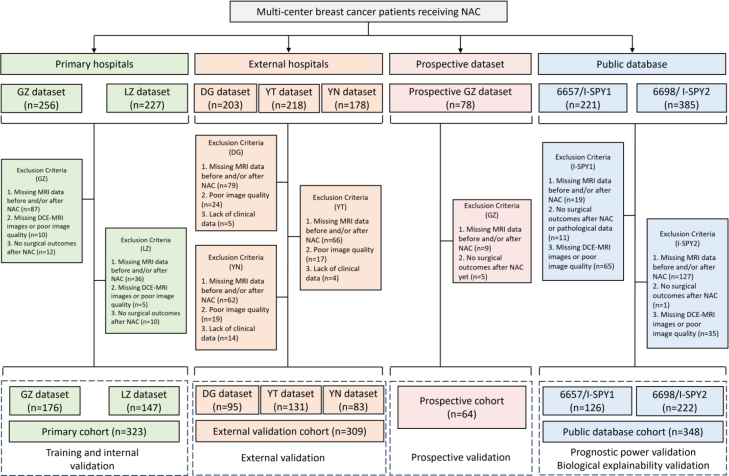
**Data curation flowchart.** This study included four cohorts: the primary cohort (PC), the external validation cohort (EVC, including EVC1, EVC2, and EVC3), the prospective validation cohort (PVC), and the public database cohort (including ACRIN 6657/I-SPY1 and ACRIN 6698/I-SPY2). Exclusion criteria were presented in this illustration. Note: NAC = neoadjuvant chemotherapy; DCE-MRI = dynamic contrast-enhanced MRI.

A total of 1044 patients from five hospitals across different regions of China and two public databases were included in this study. Detailed information on the recruitment process is provided in Fig. 1 and Supplementary A1. The retrospective data collection from the five hospitals took place from June 2018 to December 2023. For the development of the STI model, 323 patients were retrospectively recruited from Guangzhou First People’s Hospital and Liuzhou People’s Hospital as the primary cohort (PC), which was then split into training and internal validation groups in an 8:2 ratio. Additionally, 309 patients were recruited from Dongguan People’s Hospital, Yantai Yuhuangding Hospital, and Honghe Prefecture Third People’s Hospital as the external validation cohort (EVC1: n = 95, EVC2: n = 131, and EVC3: n = 83). A prospective trial was conducted from January 2024 to December 2024 with 78 subjects from Guangzhou First People’s Hospital to evaluate the clinical applicability of the STI model. Furthermore, follow-up data from 126 patients in the ACRIN 6657/I-SPY1 study were used for survival analysis, while gene pathway information from 222 patients in the ACRIN 6698/I-SPY2 study was used for biological interpretability analysis.

**Fig. 2:**
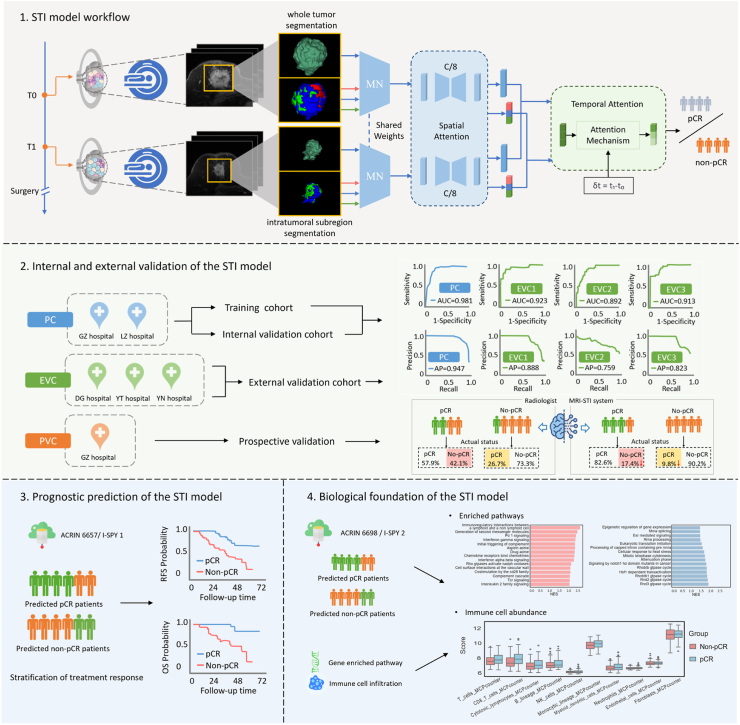
**Overview of the study.** The study consists of four key phases: (1) STI model workflow: The model processes longitudinal multi-phase MRI scans at pre-NAC (T0) and early-NAC (T1) stages, incorporating whole-tumor and subregion segmentation with spatial and temporal attention mechanisms to predict NAC response. (2) Internal and external validation: The model is trained and internally validated on PC (GZ and LZ datasets) and EVC using three independent external cohorts (DG, YT, YN datasets), followed by PVC (GZ prospective dataset). Performance metrics, including AUC and AP, evaluate model efficacy. (3) Prognostic prediction: The model’s predictive score is assessed for its correlation with RFS and OS in the ACRIN 6657/I-SPY1 dataset. (4) Biological foundation of the STI model: Pathological validation is performed using histopathological data from primary cohorts, and molecular-level validation is conducted via transcriptomic analysis from ACRIN 6698/I-SPY2 to explore associated biological mechanisms. For detailed visualization of this section, please refer to Fig. 6, Fig. 7. Note: PC = primary cohort; EVC = external validated cohorts; PVC = prospective validation cohort; RFS = recurrence-free survival; OS = overall survival.

The overall study design is illustrated in Fig. 2. The study was conducted in four phases: Phase 1: Model development; Phase 2: Multi-center validation; Phase 3: Prognostic prediction analysis; Phase 4: Biological interpretability analysis.

### Histopathology analysis and clinical outcomes

Histopathological analysis included core needle biopsy before NAC and pathological examination after surgery at the completion of NAC treatment. Immunohistochemistry (IHC) was performed before NAC to assess the status of estrogen receptor (ER), progesterone receptor (PR), human epidermal growth factor receptor-2 (HER2), and Ki-67 index. The cutoff for Ki-67 level was 14%. ER-positivity and PR-positivity were defined as more than 1% positive invasive tumor cells with nuclear staining.^32^ HER2 positivity was defined as IHC 3+ or, IHC 2+ and confirmed by fluorescence in situ hybridization (FISH) amplification.^33^ Hormone receptor (HR) positivity was defined as ER-positive and/or PR-positive. Molecular subtypes were classified into HR+/HER2−, HER2+, and HR−/HER2− (triple-negative breast cancer, TNBC). Within one month after NAC completion, the pCR status of each target tumor was determined based on surgical pathology results. pCR was defined as the absence of invasive carcinoma in the breast and axillary lymph nodes at the time of surgery (ypT0/is, ypN0), allowing for the presence of residual ductal carcinoma in situ (DCIS) (ypTis). For multicenter cohorts, a standardized pCR definition was applied.^34^ Detailed follow-up information for the ACRIN 6657/I-SPY1 cohort has been previously reported.^35^ Recurrence-free survival (RFS) and overall survival (OS) data were extracted for subsequent analysis of the model’s prognostic prediction performance.

### Image acquisition and preprocessing and tumor automatic segmentation

All MRI scans were performed within two weeks prior to NAC and after 1–2 cycles of NAC using 1.5 T or 3.0 T scanners. Detailed information on the MRI acquisition protocols and image preprocessing can be found in Supplementary A2, Supplementary Fig. S2 and Supplementary Table S1. We used a spatial–temporal transformer, previously developed in the literature,^36^ to capture dynamic changes in multi-phase DCE-MRI for automatic segmentation of three-dimensional tumor regions, with further details provided in Supplementary A3 and Supplementary Fig. S3. Subsequently, all ROIs from different centers were then reviewed by two senior radiologists (T.WJ. and G.Y., with 7 and 14 years of breast MRI experience, respectively). Any discrepancies were resolved through consensus.

### Development of a deep learning network

To enhance model generalizability, we first conducted pretraining using a contrastive learning framework on a publicly available DCE-MRI dataset, as detailed in Supplementary A4. Then the STI model based on DCE-MRI integrates a Siamese network architecture and a transformer-based multi-head temporal attention mechanism to predict breast cancer patients’ response to NAC. The Siamese network processes and compares images from two time points (T0 and T1). Simultaneously, tumor habitat segmentation is performed, enabling the model to capture spatial heterogeneity features of the tumor (Supplementary Fig. S4 and Table S2). In this framework, the multi-head transformer attention mechanism is used to capture the temporal relationships between pre- and post-treatment images, allowing the model to better understand the tumor’s dynamics over time. Details of the model development and training process can be found in Supplementary A4 and Supplementary Fig. S5.

### Clinical model and STI model integrated with clinical characteristics

In the PC cohort, univariate and multivariate logistic regression analyses were conducted to identify clinically significant predictive factors, including age, menopause status, baseline tumor long diameter (LD), clinical T stage, ER, PR, HER2, Ki-67 status, and molecular subtype. Support Vector Machine (SVM) was then employed to establish the clinical model.

For the STI model, a genetic algorithm was used to select deep features, with clinical characteristics considered during the feature selection process to form the optimal deep features for the model. These selected deep features were then combined with the chosen clinical features to construct a combined model. In this integrated model, SVM was also used as the classifier, maintaining the same model structure as the clinical model. Details regarding the development process of the STI model integrated with clinical characteristics are provided in Supplementary A5.

### Assessment of models

We used the PyTorch (version 1.13.1) and scikit-learn package (version 1.0.2) in Python 3.7.7 for model building and evaluation, respectively. To select the best hyperparameters for the model, grid search and 5-fold cross-validation were performed. To ensure the robustness of the model, a layer-wise fine-tuning approach based on pre-trained models was adopted. Training started from the bottom layers, and layers were gradually unfrozen when performance improvements were no longer significant. The model that performed best in the primary cohort was then used to test the external validation cohort.

To validate the importance of integrating spatial and longitudinal temporal information within deep networks, we compared models based on single time points, single time points with spatial information, and longitudinal temporal models. Additionally, to assess the necessity of various components in the proposed spatiotemporal interaction network for accurately predicting responses in multicenter data, we conducted ablation experiments by modifying the network architecture. Further details are provided in Supplementary A6. Finally, to evaluate the model’s stability across different subtypes, we categorized tumors into distinct molecular subtypes and clinical T stages, assessing the model’s performance within each group.

The early prediction performance for pCR was evaluated using receiver operating characteristic (ROC) curves and Precision-Recall curves, with the area under the ROC curve (AUC) and area under the Precision-Recall curve (AP) calculated accordingly. We defined pCR as a positive event. A false positive event was defined as a patient predicted by the model to have pCR, but who actually did not. In the Precision-Recall curve, precision corresponds to the positive predictive value (PPV), reflecting the proportion of patients predicted to achieve pCR who truly did so, while recall corresponds to sensitivity, representing the proportion of actual pCR patients correctly identified by the model. Precision-Recall curves are especially useful in imbalanced classification scenarios. This interpretation bridges machine learning terminology with conventional clinical diagnostic metrics, offering additional insight into model performance beyond AUC. We calculated the sensitivity, specificity, accuracy, PPV and negative predictive value (NPV) for pCR prediction. To ascertain the decision-making information captured by the STI model in predicting pCR, we utilized gradient-weighted class activation mapping (Grad-CAM) to visualize the location and distribution of decision making on the MRI images.

### Reader study and AI-assisted diagnostic evaluation

For the retrospective cohorts (PC and EVC), a reader study was conducted by three radiologists (C.SY., T.WJ., and G.Y., with 4, 7, and 14 years of breast MRI experience, respectively) to assess treatment response prediction performance compared to the STI model. The radiologists independently evaluated MRI images from T0 (pre-treatment), T0 and T1 (before and after treatment). Clinical and pathological information, including patient age, menopausal status, clinical T stage, and molecular subtype, was also provided. The radiologists, blinded to the pCR status, classified each case as pCR or non-pCR. To evaluate the potential benefit of AI assistance, a second reading was conducted after the initial assessment, where radiologists were shown the STI model’s prediction. In cases of disagreement, they could revise their judgment or retain their original decision. Final performance metrics after model-assisted decision-making were compared with both the STI model alone and radiologist-alone predictions.

For the prospective validation cohort (PVC), two radiologists (C.SY. and T.WJ., with 4 and 7 years of breast MRI experience, respectively) independently assessed MRI images from T0 and T1 to determine pCR or non-pCR. Disagreements were resolved by a third radiologist (G.Y., with 14 years of experience). Clinical and pathological information was similarly provided during evaluation for this cohort.

### Biological interpretability analysis

To investigate the molecular and pathological foundations of the STI model in stratifying treatment responses in breast cancer patients, we conducted an in-depth biological interpretability analysis at both the genomic and pathological levels.

Initially, we utilized the ACRIN 6698/I-SPY2 dataset to explore biological differences and gain a deeper understanding of the relevant biological pathways associated with NAC treatment response. Gene set enrichment analysis (GSEA) was performed using the clusterProfiler, msigdbr, and GSEAbase R packages, based on the c2.cp. reactome gene sets from the MSigDb database. This analysis aimed to identify enriched pathways between NAC treatment response groups as predicted by the STI model. Core genes showing significant differences between these pathways were identified, with enriched gene sets selected based on the following criteria: p-value < 0.05 and an absolute normalized enrichment score (NES) > 1. In addition, we conducted Gene Set Variation Analysis (GSVA) using the msigdbr package to assess variations in the gene sets. The limma package was applied to evaluate the statistical significance of these variations. For the c2.cp. reactome gene sets, the filtering criteria included a p-value < 0.05 and |logFC| > 0.1. To further investigate the relationship between deep learning features and biological pathways, we performed Spearman correlation analysis to examine significant correlations (p < 0.05) between deep features and biological functions or pathways. To assess cellular abundance within the tumor microenvironment (TME) of breast cancer samples, we applied single-sample gene set enrichment analysis (ssGSEA) to calculate the abundance of each cell subtype in individual samples. Using the “GSVA” function from the GSVA R package, we performed single-sample enrichment analysis and assigned abundance scores to different cell populations within each sample. Group comparisons were then conducted to evaluate differences in immune cell abundance between response groups.

Finally, at the pathological level, we focused on dynamic processes such as tumor proliferation, immune response, and tumor infiltration. Changes in Ki-67 expression, tumor-infiltrating lymphocytes (TILs), lymphovascular invasion (LVI), and fibrosis in the tumor bed after treatment were assessed using data from the PC cohort. The relationship between these changes and treatment response groups was evaluated, with intergroup comparisons perfor.

### Survival and statistical analysis

For the ACRIN 6657/I-SPY1 dataset, survival analysis was performed using Kaplan–Meier curves to compare survival rates based on treatment response stratification. Patients were divided into high STI score and low STI score groups based on the predicted scores generated by the STI model. The log-rank test was used to assess the significance of differences between the survival curves. Univariate Cox regression analysis was conducted to evaluate the independent association between the model’s predicted scores and both recurrence-free survival (RFS) and overall survival (OS), adjusting for clinical variables, including age, ER status, PR status, HER2 status, molecular subtype, and baseline tumor size. The likelihood ratio test was applied to determine the prognostic value of the model’s predicted scores. Hazard ratios were used as the metric for survival analysis.

For the purpose of this analysis, only the results from the external validation cohorts (EVC1, EVC2, and EVC3) were reported and discussed in the main text. The results from the PC are included in tables but are not discussed in the main text to minimize potential bias from using the same dataset for both model development and evaluation.

Baseline data of patients were analyzed using SPSS (version 20.0) and statistical packages in Python 3.12. Continuous variables are presented as mean (standard deviation), and categorical variables are described as frequencies. For intergroup comparisons, Student’s t-test or Mann–Whitney U test was used for continuous variables, while the Chi-square test or Fisher’s exact test was applied for categorical variables. The Chi-square test or Chi-square with Yates’ continuity correction was used for 2 × 2 tables, and Fisher’s Exact Test was applied to the other appropriate cases, with the two-tailed p-value calculated as double the one-tailed probability, subject to a maximum value of one. The DeLong test was used to compare differences in AUCs. All statistical tests were two-sided, with a p-value < 0.05 considered statistically significant. All analyses were conducted using R 3.4.1 and SPSS 23.0 (IBM, Armonk, NY, USA).

### Role of the funding source

The funders of this study had no roles in study design, data collection, data analysis, data interpretation, or writing of the report.

## Results

### Clinicopathological characteristics

The clinicopathological characteristics of all 1044 breast cancer patients were presented in Table 1 and Supplementary Table S3. In the PC, EVC (including EVC1, EVC2 and EVC3), and PVC, 84 (26.0%), 35 (36.8%), 38 (29.0%), 26 (31.3%), and 23 (35.9%) patients achieved pCR after NAC treatment, respectively. Significant differences in ER, PR, and HER2 status were observed between the pCR and non-pCR groups in the PC and most external validation cohorts (EVC1, EVC2), with all p-values < 0.05. In EVC3, although ER and PR status did not reach conventional statistical significance (p = 0.064 and 0.060, respectively), a similar trend was observed. Baseline long diameter (LD) showed a significant difference only in the EVC 3 dataset (p = 0.015), while no significant differences were observed in other datasets (Table 1). The public datasets show that 30 (23.8%) out of 126 patients in the ACRIN 6657/I-SPY 1 cohort and 54 (24.3%) out of 222 patients in the ACRIN 6698/I-SPY 2 cohort achieved pCR, and detailed clinicopathological characteristics are summarized in Supplementary Table S3.

**Table 1:** Baseline clinicopathological characteristics of the patients from hospital in different cohorts.

Characteristics	Primary cohort (N = 323)			External validation cohort 1 (N = 95)			External validation cohort 2 (N = 131)			External validation cohort 3 (N = 83)			Prospective validation cohort (N = 64)		
	pCR (n = 84)	non-pCR (n = 239)	p value	pCR (n = 35)	non-pCR (n = 60)	p value	pCR (n = 38)	non-pCR (n = 93)	p value	pCR (n = 26)	non-pCR (n = 57)	p value	pCR (n = 23)	non-pCR (n = 41)	p value
**Age**	52.2 (9.1)	51.5 (10.4)	0.56	51.0 (9.0)	48.9 (11.2)	0.35	50.7 (8.7)	50.8 (8.5)	0.96	51.7 (9.9)	51.6 (8.3)	0.96	49.5 (9.5)	54.2 (9.8)	0.070
**Menopause**			0.15			0.37^a^			0.96^a^			0.097^a^			0.78^a^
Postmenopausal	53	127		25	36		22	56		19	29		13	26	
Premenopausal	31	112		10	24		16	37		7	28		10	15	
**LD at baseline (mm)**	36.4 (18.1)	38.7 (19.0)	0.34	33.7 (14.8)	38.1 (17.3)	0.22	36.5 (15.2)	39.4 (16.7)	0.36	30.1 (14.1)	40.2 (18.2)	0.015	37.0 (16.2)	40.9 (16.1)	0.36
**Clinical T stage**			0.30			0.14^a^			0.18^a^			0.13^a^			0.45^a^
T1–T2	61	159		31	44		29	58		19	30		19	29	
T3–T4	23	80		4	16		9	35		7	27		4	12	
**ER status**			<0.0001			0.0020^a^			0.0025^a^			0.064^a^			0.0007^a^
Positive	32	185		15	46		18	71		12	40		8	33	
Negative	52	54		20	14		20	22		14	17		15	8	
**PR status**			<0.0001			0.010^a^			<0.0001^a^			0.060^a^			0.0058^a^
Positive	29	168		17	46		11	71		9	34		5	25	
Negative	55	71		18	14		27	22		17	23		18	16	
**HER2 status**			<0.0001			<0.0001^a^			0.0001^a^			0.0049^a^			0.0087^a^
Positive	69	68		29	20		32	43		17	17		14	10	
Negative	15	171		6	40		6	50		9	40		9	31	
**Ki-67 status**			0.35			0.26^b^			0.33^b^			0.73^b^			0.22^b^
Positive	79	217		34	53		36	81		23	47		21	31	
Negative	5	22		1	7		2	12		3	10		2	10	
**Molecular subtype**			<0.0001			<0.0001			<0.0001			0.0093			<0.0001
HR+/HER2−	8	145		3	31		1	44		7	30		1	26	
HER2+	69	67		29	20		32	43		17	17		14	10	
HR−/HER2− (TN)	7	27		3	9		5	6		2	10		8	5	

Data are presented as number of patients, except for age and LD at baseline (mean [SD]).

pCR: pathological complete response; ER: estrogen receptor; PR: progesterone receptor; HER2: human epidermal growth factor receptor; HR: hormone receptor; SD: standard deviation.

a For Pearson’s chi-squared test, Yates’ continuity correction was applied to 2 × 2 tables.

b For Fisher’s Exact Test, two-tailed p-values were calculated by doubling the one-tailed exact probability, subject to a maximum value of one.

### Predictive performance of the STI model

The performance of various deep learning models integrating spatial and longitudinal temporal information for predicting pCR to NAC was comprehensively evaluated across different cohorts.

**Fig. 3:**
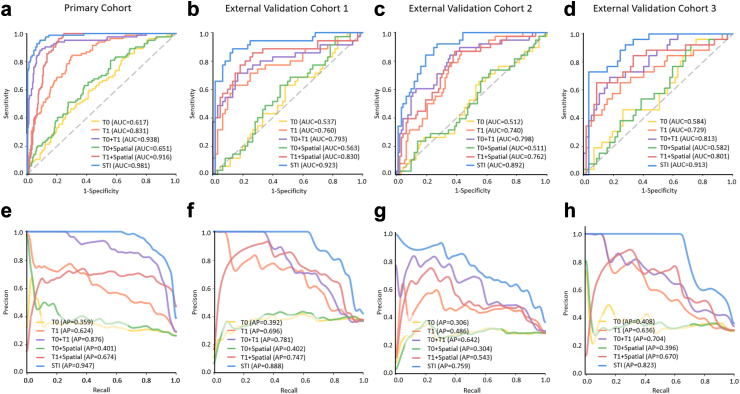
**Predictive performances of the different models in the PC and EVC after NAC.** (a–d) Plots show the ROC curves of six models (T0 model, T1 model, T0 + T1 model, T0 + Spatial model, T1 + Spatial model, STI model) in PC (a), EVC1 (b), EVC2 (c) and EVC3 (d), respectively. (e–h) Plots show the Precision-Recall curves of six models (T0 model, T1 model, T0 + T1 model, T0 + Spatial model, T1 + Spatial model, STI model) in PC (e), EVC1 (f), EVC2 (g) and EVC3 (h), respectively. Note: PC = primary cohort; EVC = external validated cohorts; pCR = Pathological complete response; STI = Spatiotemporal interaction model.

The STI model demonstrated superior performance in predicting treatment response in breast cancer. The AUCs for the STI model were 0.923 (95% CI: 0.859–0.987), 0.892 (95% CI: 0.821–0.963), and 0.913 (95% CI: 0.835–0.991), while APs were 0.888 (95% CI: 0.784–0.964), 0.759 (95% CI: 0.611–0.882), and 0.823 (95% CI: 0.682–0.938), across the EVC1, EVC2, and EVC3, respectively. Satisfactory sensitivity and specificity were also observed (Table 2, Fig. 3, Supplementary Table S4).

**Table 2:** Performances of deep learning models combined with different time and space for predicting pCR to NAC in different cohorts.

Model	Cohorts	AUC (95% CI)	AP (95% CI)	Accuracy (95% CI)	Sensitivity (95% CI)	Specificity (95% CI)	PPV (95% CI)	NPV (95% CI)
T0 model	**PC**	0.617 (0.545–0.689)	0.359 (0.285–0.466)	0.625 (0.570–0.678)	0.476 (0.366–0.588)	0.678 (0.615–0.737)	0.342 (0.257–0.435)	0.786 (0.724–0.840)
	**EVC total**	0.537 (0.468–0.607)	0.343 (0.285–0.439)	0.518 (0.461–0.575)	0.485 (0.383–0.587)	0.533 (0.463–0.602)	0.329 (0.253–0.411)	0.687 (0.610–0.757)
	EVC 1	0.537 (0.416–0.659)	0.392 (0.295–0.560)	0.537 (0.432–0.640)	0.486 (0.314–0.660)	0.567 (0.432–0.694)	0.395 (0.250–0.556)	0.654 (0.509–0.780)
	EVC 2	0.512 (0.402–0.621)	0.306 (0.220–0.443)	0.534 (0.445–0.622)	0.368 (0.218–0.540)	0.602 (0.495–0.702)	0.275 (0.159–0.417)	0.700 (0.587–0.797)
	EVC 3	0.584 (0.449–0.719)	0.408 (0.272–0.587)	0.470 (0.359–0.583)	0.654 (0.443–0.828)	0.386 (0.260–0.524)	0.327 (0.203–0.471)	0.710 (0.520–0.858)
T1 model	**PC**	0.831 (0.774–0.888)	0.624 (0.518–0.735)	0.783 (0.734–0.827)	0.440 (0.332–0.553)	0.904 (0.859–0.938)	0.617 (0.482–0.739)	0.821 (0.770–0.866)
	**EVC total**	0.732 (0.669–0.795)	0.573 (0.482–0.681)	0.738 (0.685–0.786)	0.465 (0.364–0.568)	0.867 (0.813–0.910)	0.622 (0.501–0.732)	0.774 (0.716–0.826)
	EVC 1	0.760 (0.655–0.865)	0.696 (0.522–0.842)	0.758 (0.659–0.840)	0.629 (0.449–0.785)	0.833 (0.715–0.917)	0.688 (0.500–0.839)	0.794 (0.673–0.885)
	EVC 2	0.740 (0.640–0.840)	0.486 (0.362–0.682)	0.725 (0.640–0.800)	0.237 (0.114–0.402)	0.925 (0.851–0.969)	0.562 (0.299–0.802)	0.748 (0.658–0.824)
	EVC 3	0.729 (0.606–0.853)	0.636 (0.447–0.797)	0.735 (0.627–0.826)	0.577 (0.369–0.766)	0.807 (0.681–0.900)	0.577 (0.369–0.766)	0.807 (0.681–0.900)
T0 + T1 model	**PC**	0.938 (0.901–0.975)	0.876 (0.812–0.924)	0.901 (0.863–0.931)	0.798 (0.696–0.877)	0.937 (0.899–0.964)	0.817 (0.716–0.894)	0.929 (0.889–0.958)
	**EVC total**	0.792 (0.734–0.850)	0.690 (0.599–0.776)	0.767 (0.716–0.813)	0.596 (0.493–0.693)	0.848 (0.792–0.893)	0.648 (0.541–0.746)	0.817 (0.759–0.866)
	EVC 1	0.793 (0.693–0.892)	0.781 (0.663–0.875)	0.758 (0.659–0.840)	0.743 (0.567–0.875)	0.767 (0.640–0.866)	0.650 (0.483–0.794)	0.836 (0.712–0.922)
	EVC 2	0.798 (0.706–0.890)	0.642 (0.495–0.800)	0.771 (0.689–0.840)	0.421 (0.263–0.592)	0.914 (0.838–0.962)	0.667 (0.447–0.844)	0.794 (0.705–0.866)
	EVC 3	0.813 (0.704–0.922)	0.704 (0.519–0.852)	0.771 (0.666–0.856)	0.654 (0.443–0.828)	0.825 (0.701–0.913)	0.630 (0.424–0.806)	0.839 (0.717–0.924)
T0 + spatial model	**PC**	0.651 (0.580–0.722)	0.401 (0.317–0.511)	0.666 (0.611–0.717)	0.512 (0.400–0.623)	0.720 (0.658–0.776)	0.391 (0.299–0.489)	0.808 (0.748–0.858)
	**EVC total**	0.544 (0.475–0.614)	0.350 (0.284–0.435)	0.528 (0.470–0.584)	0.465 (0.364–0.568)	0.557 (0.487–0.625)	0.331 (0.254–0.416)	0.688 (0.613–0.757)
	EVC 1	0.563 (0.442–0.684)	0.402 (0.291–0.557)	0.558 (0.452–0.660)	0.486 (0.314–0.660)	0.600 (0.465–0.724)	0.415 (0.263–0.579)	0.667 (0.525–0.789)
	EVC 2	0.511 (0.401–0.620)	0.304 (0.215–0.444)	0.527 (0.438–0.615)	0.368 (0.218–0.540)	0.591 (0.485–0.692)	0.269 (0.156–0.410)	0.696 (0.582–0.795)
	EVC 3	0.582 (0.447–0.717)	0.396 (0.270–0.584)	0.494 (0.382–0.606)	0.577 (0.369–0.766)	0.456 (0.324–0.593)	0.326 (0.195–0.480)	0.703 (0.530–0.841)
T1 + spatial model	**PC**	0.916 (0.873–0.958)	0.674 (0.573–0.795)	0.848 (0.804–0.886)	0.798 (0.696–0.877)	0.866 (0.816–0.907)	0.677 (0.575–0.767)	0.924 (0.881–0.955)
	**EVC total**	0.791 (0.733–0.849)	0.629 (0.533–0.740)	0.748 (0.695–0.795)	0.566 (0.462–0.665)	0.833 (0.776–0.881)	0.615 (0.508–0.716)	0.803 (0.744–0.853)
	EVC 1	0.830 (0.738–0.922)	0.747 (0.595–0.902)	0.779 (0.682–0.858)	0.629 (0.449–0.785)	0.867 (0.754–0.941)	0.733 (0.541–0.877)	0.800 (0.682–0.889)
	EVC 2	0.762 (0.665–0.859)	0.543 (0.396–0.727)	0.733 (0.648–0.806)	0.447 (0.286–0.617)	0.849 (0.760–0.915)	0.548 (0.360–0.727)	0.790 (0.697–0.865)
	EVC 3	0.801 (0.690–0.912)	0.670 (0.493–0.860)	0.735 (0.627–0.826)	0.654 (0.443–0.828)	0.772 (0.642–0.873)	0.567 (0.374–0.745)	0.830 (0.702–0.919)
STI model	**PC**	0.981 (0.960–1.002)	0.947 (0.901–0.979)	0.907 (0.870–0.936)	0.952 (0.883–0.987)	0.891 (0.845–0.928)	0.755 (0.662–0.833)	0.982 (0.953–0.995)
	**EVC total**	0.898 (0.855–0.941)	0.807 (0.733–0.874)	0.806 (0.757–0.848)	0.859 (0.774–0.920)	0.781 (0.719–0.835)	0.649 (0.561–0.730)	0.921 (0.872–0.956)
	EVC 1	0.923 (0.859–0.987)	0.888 (0.784–0.964)	0.821 (0.729–0.892)	0.943 (0.808–0.993)	0.750 (0.621–0.853)	0.688 (0.537–0.813)	0.957 (0.855–0.995)
	EVC 2	0.892 (0.821–0.963)	0.759 (0.611–0.882)	0.809 (0.731–0.873)	0.763 (0.598–0.886)	0.828 (0.736–0.898)	0.644 (0.488–0.781)	0.895 (0.811–0.951)
	EVC 3	0.913 (0.835–0.991)	0.823 (0.682–0.938)	0.783 (0.679–0.866)	0.885 (0.698–0.976)	0.737 (0.603–0.845)	0.605 (0.434–0.760)	0.933 (0.817–0.986)

T0 model: Based solely on features from the whole tumor at the pre-treatment timepoint (T0); T1 model is defined similarly with T0 model.

T0 + Spatial model: Combines whole-tumor features and spatial heterogeneity features extracted from tumor subregions at T0; T1 + Spatial model is defined similarly with T0 + Spatial model.

95% confidence intervals for AUC and AP were calculated using the DeLong method. 95% confidence intervals for sensitivity, specificity, accuracy, PPV, and NPV were calculated using the exact Clopper-Pearson method.

pCR, pathological complete response; AUC: area under the receiver operating characteristics curve; AP: area under the P-R(precision-recall) cruve; PPV: positive predictive value; NPV: negative predictive value; PC, primary cohort; EVC, external validation cohort; 95% CI, 95% confidence interval; STI model: Spatiotemporal interaction model.

In contrast, the T0 model, which utilizes a single time point, exhibited the lowest performance across all datasets, with AUCs ranging from 0.512 to 0.584 and APs from 0.306 to 0.408. The addition of spatial information (T0 + Spatial model) did not significantly improve performance, with AUCs ranging from 0.511 to 0.582 and APs from 0.304 to 0.402. Compared with the T0 model, the T0 + Spatial model improved the AUC by 1.3% (p = 0.43) in the EVC total. The T1 model, which incorporates a single time point in the early post treatment, showed improved predictive performance, with AUCs ranging from 0.729 to 0.760 and APs from 0.486 to 0.696. Adding spatial information (T1 + Spatial model) led to a further noticeable improvement, with AUCs of 0.762–0.830 and APs of 0.543–0.747. Compared to the T1 model, the T1 + Spatial model increased the AUC by 8.1% (p = 0.037) in the EVC total. The T0 + T1 model, which incorporates longitudinal temporal data, showed further improvements, with AUCs of 0.793–0.813 and APs of 0.642–0.781. Compared to the T0 model and T1 model, the T0 + T1 model increased the AUC by 47.5% (p < 0.0001) and 8.2% (p = 0.038) respectively in the EVC total. Building upon the T0 + T1 model, the STI model, which integrates both spatial and temporal information, achieved the highest predictive performance. Compared to the T0 + T1 model, the STI model further increased the AUC by 13.4% (p < 0.0001) in the EVC total. Compared to the T0 + Spatial and T1 + models, the STI model integrates spatiotemporal features, resulting in 65.1% (p < 0.0001) and 13.5% (p = 0.0001) improvement in AUC for predicting NAC response in the EVC total (Supplementary Fig. S6).

To better assess the role of spatial heterogeneity, we introduced the T0 + T1 only spatial model, which utilizes spatial features from tumor subregions at both T0 and T1, excluding whole-tumor features. This model was evaluated across the entire cohort. As shown in Supplementary Table S5, the T0 + T1 only spatial model achieved an AUC of 0.810 (95% CI: 0.754–0.866) for identifying pCR in the total EVC. The STI model improved the AUC by 10.9% compared to the T0 + T1 only spatial model (p = 0.0010).

### Subgroup analysis

To further investigate the robustness and generalizability of the STI model across different breast cancer subgroups, we conducted subgroup analyses based on molecular subtypes and clinical T stages.

The STI model demonstrated consistently high predictive performance across different molecular subtypes, including HR+/HER2−, HER2+, and triple-negative breast cancer (TNBC) (Table 3, Supplementary Table S6). In the HR+/HER2− subgroup, the model achieved the highest predictive performance, with an AUC of 0.944 (95% CI: 0.849–1.040) in the EVC total. The accuracy remained high across all validation cohorts, confirming the model’s effectiveness in this subgroup. For HER2+ breast cancer, the STI model maintained strong predictive ability, achieving an AUC of 0.857 (95% CI: 0.796–0.916) in the EVC total. In the TNBC subgroup, the STI model also exhibited excellent predictive power, with an AUC of 0.920 (95% CI: 0.798–1.041) in the EVC total.

**Table 3:** Performances of STI models for predicting pCR to NAC in various molecular subtypes and clinical T stage.

Molecular subtype	Cohorts	AUC (95% CI)	Accuracy (95% CI)	Sensitivity (95% CI)	Specificity (95% CI)	PPV (95% CI)	NPV (95% CI)
HR+/HER2−	**PC**	0.994 (0.956–1.032)	0.954 (0.907–0.981)	1.000 (0.631–1.000)	0.951 (0.902–0.980)	0.533 (0.266–0.787)	1.000 (0.973–1.000)
	**EVC total**	0.944 (0.849–1.040)	0.858 (0.780–0.917)	0.818 (0.482–0.977)	0.863 (0.780–0.923)	0.391 (0.197–0.615)	0.978 (0.922–0.997)
	EVC 1	0.995 (0.935–1.054)	0.853 (0.689–0.950)	1.000 (0.292–1.000)	0.839 (0.663–0.945)	0.375 (0.085–0.755)	1.000 (0.868–1.000)
	EVC 2	0.989 (0.841–1.137)	0.911 (0.788–0.975)	1.000 (0.025–1.000)	0.909 (0.783–0.975)	0.200 (0.005–0.716)	1.000 (0.912–1.000)
	EVC 3	0.894 (0.731–1.057)	0.794 (0.621–0.913)	0.714 (0.290–0.963)	0.815 (0.619–0.937)	0.500 (0.187–0.813)	0.917 (0.730–0.990)
HER2+	**PC**	0.963 (0.930–0.995)	0.905 (0.843–0.949)	0.942 (0.858–0.984)	0.868 (0.764–0.938)	0.878 (0.782–0.943)	0.937 (0.845–0.982)
	**EVC total**	0.857 (0.796–0.916)	0.766 (0.692–0.829)	0.910 (0.824–0.963)	0.625 (0.510–0.731)	0.703 (0.604–0.790)	0.877 (0.763–0.949)
	EVC 1	0.883 (0.789–0.977)	0.776 (0.634–0.882)	0.931 (0.772–0.992)	0.550 (0.315–0.769)	0.750 (0.578–0.879)	0.846 (0.546–0.981)
	EVC 2	0.860 (0.769–0.950)	0.787 (0.677–0.873)	0.875 (0.710–0.965)	0.721 (0.563–0.847)	0.700 (0.535–0.834)	0.886 (0.733–0.968)
	EVC 3	0.851 (0.719–0.984)	0.706 (0.525–0.849)	0.941 (0.713–0.999)	0.471 (0.230–0.722)	0.640 (0.425–0.820)	0.889 (0.518–0.997)
HR−/HER2− (TNBC)	**PC**	0.979 (0.902–1.056)	0.912 (0.763–0.981)	1.000 (0.590–1.000)	0.889 (0.708–0.976)	0.700 (0.348–0.933)	1.000 (0.858–1.000)
	**EVC total**	0.920 (0.798–1.041)	0.868 (0.719–0.956)	0.800 (0.444–0.975)	0.893 (0.718–0.977)	0.727 (0.390–0.940)	0.926 (0.757–0.991)
	EVC 1	0.963 (0.8037–1.122)	0.833 (0.516–0.979)	1.000 (0.292–1.000)	0.778 (0.400–0.972)	0.600 (0.147–0.947)	1.000 (0.590–1.000)
	EVC 2	0.867 (0.630–1.104)	0.818 (0.482–0.977)	0.600 (0.147–0.947)	1.000 (0.541–1.000)	1.000 (0.292–1.000)	0.750 (0.349–0.968)
	EVC 3	1.000 (1.000–1.000)	0.933 (0.681–0.998)	1.000 (0.158–1.000)	0.923 (0.640–0.998)	0.667 (0.094–0.992)	1.000 (0.735–1.000)
**Clinical T stage**							
T1–T2	**PC**	0.974 (0.945–1.002)	0.914 (0.868–0.947)	0.902 (0.798–0.963)	0.918 (0.864–0.956)	0.809 (0.695–0.894)	0.961 (0.916–0.985)
	**EVC total**	0.871 (0.817–0.925)	0.778 (0.716–0.832)	0.850 (0.753–0.920)	0.735 (0.651–0.808)	0.660 (0.560–0.751)	0.890 (0.816–0.942)
	EVC 1	0.911 (0.838–0.985)	0.813 (0.707–0.894)	0.935 (0.786–0.992)	0.727 (0.572–0.850)	0.707 (0.545–0.839)	0.941 (0.803–0.993)
	EVC 2	0.856 (0.764–0.948)	0.773 (0.671–0.855)	0.733 (0.541–0.877)	0.793 (0.666–0.888)	0.647 (0.465–0.803)	0.852 (0.729–0.934)
	EVC 3	0.880 (0.771–0.988)	0.735 (0.589–0.851)	0.895 (0.669–0.987)	0.633 (0.439–0.801)	0.607 (0.406–0.785)	0.905 (0.696–0.988)
T3–T4	**PC**	0.993 (0.968–1.018)	0.932 (0.865–0.972)	1.000 (0.852–1.000)	0.912 (0.828–0.964)	0.767 (0.577–0.901)	1.000 (0.951–1.000)
	**EVC total**	0.949 (0.879–1.019)	0.866 (0.782–0.927)	0.895 (0.669–0.987)	0.859 (0.762–0.927)	0.607 (0.406–0.785)	0.971 (0.899–0.996)
	EVC 1	0.969 (0.845–1.093)	0.850 (0.621–0.968)	1.000 (0.398–1.000)	0.812 (0.544–0.960)	0.571 (0.184–0.901)	1.000 (0.753–1.000)
	EVC 2	0.964 (0.872–1.056)	0.884 (0.749–0.961)	0.875 (0.473–0.997)	0.886 (0.733–0.968)	0.636 (0.308–0.891)	0.969 (0.838–0.999)
	EVC 3	0.931 (0.797–1.066)	0.853 (0.689–0.950)	0.857 (0.421–0.996)	0.852 (0.663–0.958)	0.600 (0.262–0.878)	0.958 (0.789–0.999)

95% confidence intervals for AUC were calculated using the DeLong method. 95% confidence intervals for sensitivity, specificity, accuracy, PPV, and NPV were calculated using the exact Clopper-Pearson method.

pCR: pathological complete response; AUC: area under the receiver operating characteristics curve; PPV: positive predictive value; NPV: negative predictive value; HR: hormone receptor; HER2: human epidermal growth factor receptor; PC, primary cohort; EVC, external validation cohort; 95% CI, 95% confidence interval; STI model: Spatiotemporal interaction model.

The predictive performance of the STI model was further evaluated across different clinical T stages (T1–T2 and T3–T4) (Table 3, Supplementary Table S6). The model demonstrated strong predictive capability for both early-stage and advanced-stage tumors. For T1–T2 tumors, the model achieved an AUC of 0.871 (95% CI: 0.817–0.925) in the EVC total cohort. Sensitivity and specificity remained satisfactory, indicating that the model is well-suited for predicting pCR in patients with smaller tumors. For T3–T4 tumors, the STI model exhibited even higher AUC values, with an AUC of 0.949 (95% CI: 0.879–1.019) in the EVC total cohort.

### Ablation studies for STI model

To evaluate the necessity of different components in the STI model, we conducted ablation experiments by separately removing the spatial attention module and the temporal attention module.

As shown in Table 4, Supplementary Table S7 and Supplementary Fig. S7, eliminating the spatial attention module resulted in a decline in predictive performance across all cohorts. In the EVC total, it decreased from 0.898 to 0.801 (p = 0.0001). These findings indicate that spatial attention plays a crucial role in capturing key tumor-related spatial features, thereby enhancing the model’s predictive accuracy.

**Table 4:** The ablation studies of no spatial or time attention for STI model in different cohorts.

Model	Cohorts	AUC (95% CI)	Accuracy (95% CI)	Sensitivity (95% CI)	Specificity (95% CI)	PPV (95% CI)	NPV (95% CI)
**No spatial attention**	**PC**	0.845 (0.790–0.900)	0.777 (0.728–0.821)	0.679 (0.568–0.776)	0.812 (0.756–0.859)	0.559 (0.457–0.657)	0.878 (0.827–0.918)
	**EVC total**	0.801 (0.744–0.858)	0.706 (0.651–0.756)	0.768 (0.672–0.847)	0.676 (0.608–0.739)	0.528 (0.443–0.611)	0.861 (0.798–0.910)
	EVC 1	0.757 (0.652–0.863)	0.632 (0.526–0.728)	0.829 (0.664–0.934)	0.517 (0.384–0.648)	0.500 (0.366–0.634)	0.838 (0.680–0.938)
	EVC 2	0.824 (0.737–0.911)	0.740 (0.657–0.813)	0.632 (0.460–0.782)	0.785 (0.688–0.863)	0.545 (0.388–0.696)	0.839 (0.745–0.909)
	EVC 3	0.842 (0.740–0.944)	0.735 (0.627–0.826)	0.885 (0.698–0.976)	0.667 (0.529–0.786)	0.548 (0.387–0.702)	0.927 (0.801–0.985)
**No time attention**	**PC**	0.860 (0.807–0.913)	0.845 (0.801–0.883)	0.643 (0.531–0.744)	0.916 (0.874–0.948)	0.730 (0.614–0.826)	0.880 (0.832–0.917)
	**EVC total**	0.793 (0.735–0.851)	0.751 (0.699–0.798)	0.515 (0.413–0.617)	0.862 (0.808–0.906)	0.638 (0.522–0.742)	0.790 (0.732–0.841)
	EVC 1	0.812 (0.716–0.908)	0.737 (0.636–0.822)	0.571 (0.394–0.737)	0.833 (0.715–0.917)	0.667 (0.472–0.827)	0.769 (0.648–0.865)
	EVC 2	0.750 (0.651–0.849)	0.702 (0.616–0.779)	0.342 (0.196–0.514)	0.849 (0.760–0.915)	0.481 (0.287–0.681)	0.760 (0.666–0.838)
	EVC 3	0.798 (0.685–0.910)	0.843 (0.747–0.914)	0.692 (0.482–0.857)	0.912 (0.807–0.971)	0.783 (0.563–0.925)	0.867 (0.754–0.941)

95% confidence intervals for AUC were calculated using the DeLong method. 95% confidence intervals for sensitivity, specificity, accuracy, PPV, and NPV were calculated using the exact Clopper-Pearson method.

AUC: area under the receiver operating characteristics curve; PPV: positive predictive value; NPV: negative predictive value; PC, primary cohort; EVC, external validation cohort; 95% CI, 95% confidence interval; STI model: Spatiotemporal interaction model.

Similarly, the removal of the temporal attention module also led to a decline in performance. The AUC in the EVC total, it dropped from 0.898 to 0.793 (p < 0.0001). Compared to the spatial ablation scenario, the reduction in sensitivity was more pronounced, especially in the external validation cohort, where it fell from 0.859 (85/99) to 0.515 (51/99). These results highlight the importance of temporal attention in utilizing longitudinal information and tracking tumor progression over time.

Comparing both ablation settings with the complete STI model, we observed that the model incorporating both spatial and temporal attention consistently outperformed the ablated versions in both the PC and EVC. These findings underscore the synergistic effect of integrating spatiotemporal features in improving the model’s ability to predict pCR response effectively.

In addition, we performed ablations on preprocessing and pretraining to assess their impact (Supplementary Tables S8 and S9). Removing preprocessing reduced the AUC in the EVC total from 0.923 to 0.803 (p < 0.0001), highlighting its role in mitigating scanner-related variability. Similarly, removing pretraining caused the AUC to drop from 0.923 to 0.797 (p = 0.0001), suggesting that pretraining improves the model’s generalization and performance on external datasets.

### Model visualization

**Fig. 4:**
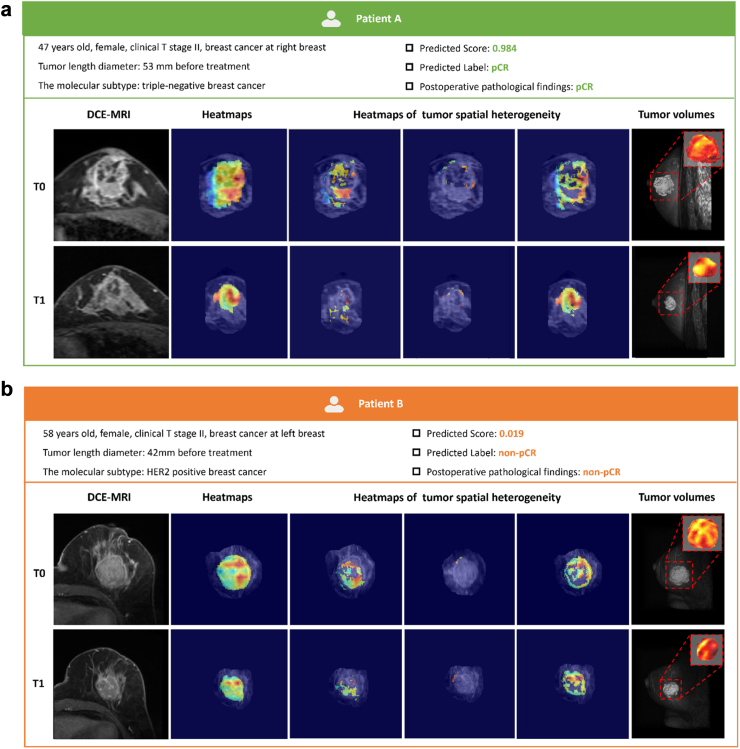
**Visualisation of two examples.** Patient A (a) achieved pathological complete response (pCR), and Patient B (b) achieved non-pCR after NAC. For each patient, the columns display the following: (1) DCE-MRI images at pre-treatment (NAC-T0) and post-treatment (NAC-T1) timepoints, (2) heatmaps (whole tumor, subregion 1, subregion 2, subregion 3) showing the spatial distribution of tumor characteristics across whole tumor and three regions, and (3) tumor volume reconstructions with corresponding heatmaps illustrating changes over time. The STI model correctly predicted the outcomes for both patients, with Patient A receiving a pCR diagnosis and Patient B a non-pCR diagnosis, as indicated by the predicted scores (0.984 and 0.019, respectively).

To determine the decision-making information captured by the STI model in predicting pCR, we utilized Grad-CAM to visualize the locations and distribution of the model’s decisions on MRI images. The Grad-CAM technique visualizes pixel weight distributions through different colors, highlighting the differences between the pCR and non-pCR groups in tumor MRI images at pre-treatment (T0) and post-treatment (T1) time points. To further understand the STI model’s decision-making, we selected two patients from the primary cohort for observation. Notably, the STI model generated attention on the three subregions of the tumor, each corresponding to distinct spatial features. These visualizations illustrate how the STI model highlights intra-tumoral spatial heterogeneity and tracks temporal changes, accurately predicting treatment outcomes for the patients (Fig. 4).

### Comparison with the clinical model and STI model combining with clinical data

As shown in Table 5, Supplementary Table S10 and Supplementary Fig. S8, the clinical model (which incorporates ER, PR, HER2, and molecular subtype) exhibited poorer predictive performance across all cohorts. Specifically, the AUC of the clinical model for the total EVC was 0.661 (95% CI: 0.594–0.727), which was significantly lower than that of the STI model (p < 0.05).

**Table 5:** The performance of clinical model and STI model combining with clinical data.

Model	Cohorts	AUC (95% CI)	Accuracy (95% CI)	Sensitivity (95% CI)	Specificity (95% CI)	PPV (95% CI)	NPV (95% CI)	p value^a^
Clinical model	**PC**	0.686 (0.615–0.756)	0.810 (0.763–0.851)	0.659 (0.546–0.760)	0.862 (0.812–0.903)	0.621 (0.510–0.723)	0.880 (0.832–0.919)	<0.0001
	**EVC total**	0.661 (0.594–0.727)	0.770 (0.719–0.815)	0.604 (0.502–0.700)	0.849 (0.794–0.894)	0.656 (0.550–0.751)	0.818 (0.761–0.867)	<0.0001
	EVC 1	0.612 (0.493–0.732)	0.758 (0.659–0.840)	0.514 (0.340–0.686)	0.900 (0.795–0.962)	0.750 (0.533–0.902)	0.761 (0.645–0.854)	<0.0001
	EVC 2	0.649 (0.544–0.754)	0.770 (0.690–0.838)	0.650 (0.483–0.794)	0.821 (0.729–0.892)	0.605 (0.444–0.750)	0.848 (0.758–0.914)	0.0002
	EVC 3	0.717 (0.592–0.843)	0.783 (0.679–0.866)	0.654 (0.443–0.828)	0.842 (0.721–0.925)	0.654 (0.443–0.828)	0.842 (0.721–0.925)	0.012
STI model + Clinical data	**PC**	0.988 (0.972–1.000)	0.931 (0.898–0.957)	0.951 (0.880–0.987)	0.925 (0.884–0.955)	0.812 (0.720–0.885)	0.982 (0.955–0.995)	0.20
	**EVC total**	0.902 (0.860–0.944)	0.812 (0.764–0.854)	0.838 (0.751–0.905)	0.800 (0.739–0.852)	0.664 (0.574–0.746)	0.913 (0.863–0.949)	0.78
	EVC 1	0.893 (0.808–0.979)	0.800 (0.705–0.875)	0.886 (0.733–0.968)	0.750 (0.621–0.853)	0.674 (0.520–0.805)	0.918 (0.804–0.977)	0.88
	EVC 2	0.911 (0.846–0.976)	0.832 (0.757–0.892)	0.816 (0.657–0.923)	0.839 (0.748–0.907)	0.674 (0.520–0.805)	0.918 (0.838–0.966)	0.34
	EVC 3	0.919 (0.853–0.985)	0.807 (0.706–0.886)	0.846 (0.651–0.956)	0.789 (0.661–0.886)	0.647 (0.465–0.803)	0.918 (0.804–0.977)	0.44

95% confidence intervals for AUC were calculated using the DeLong method. 95% confidence intervals for sensitivity, specificity, accuracy, PPV, and NPV were calculated using the exact Clopper-Pearson method.

AUC: area under the receiver operating characteristics curve; PPV: positive predictive value; NPV: negative predictive value; PC, primary cohort; EVC, external validation cohort; 95% CI, 95% confidence interval; STI model: Spatiotemporal interaction model.

a Indicates compared with AUC of STI model shown in Table 2.

Interestingly, when clinical information was added to the STI model, the performance did not show significant improvement. The AUC of the STI model combining with clinical data in the EVC total, the AUC was 0.902 (95% CI: 0.860–0.944). There was no statistically significant difference in AUC between the STI model and the STI model combining with clinical data (p > 0.05).

### Comparison of STI model, radiologists and radiologists with AI assistance

**Fig. 5:**
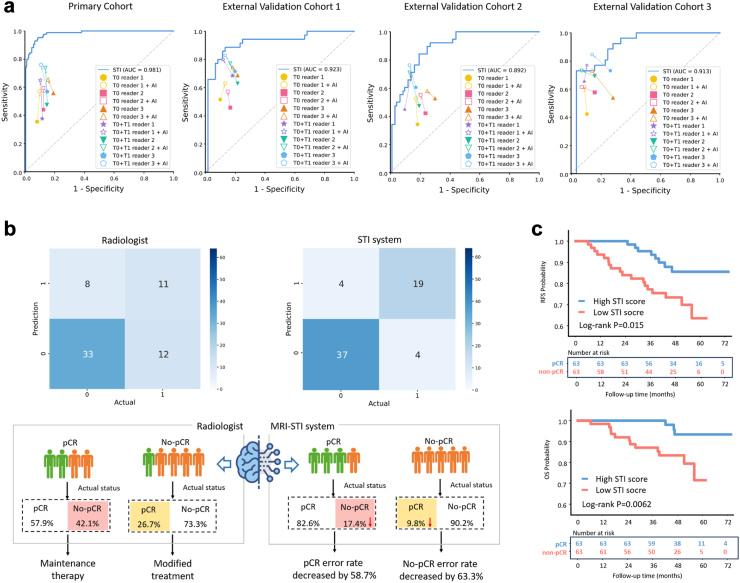
**Performance and prognostic value of the STI model compared to radiologists’ evaluations in predicting NAC response.** (a) ROC curves comparing the performance of the STI model (blue) with the assessments of three radiologists using T0 and T0 + T1 MRI across the primary cohort and external validation cohorts. Each symbol represents the sensitivity and specificity of an individual radiologist’s prediction. The performance of radiologists is compared with and without AI assistance. In the AI assisted reading experiment, radiologists were provided with the STI model’s prediction results for each case and could revise their diagnoses accordingly. (b) Performance Comparison of Radiologists vs. STI Model, in a prospective validation cohort. The matrices show the true vs. predicted classifications for each model (top). Decision tree comparison between radiologists and the STI system for predicting pCR or non-pCR outcomes (bottom). (c) Kaplan–Meier survival curves illustrating RFS (top) and OS (bottom) based on STI model-predicted NAC response. Note: PC = primary cohort; EVC = external validated cohorts; STI = Spatiotemporal interaction model; ROC = Receiver operating characteristic; RFS = recurrence-free survival; OS = overall survival.

To further explore the potential complementarity between the STI model and radiologists, we compared the prediction performance of the STI model, individual radiologists, and radiologists assisted by the model across the external validation cohorts (Fig. 5a). In this study, the STI model outperformed radiologists in prediction performance across EVC cohorts. In the internal comparison among radiologists, we also observed differences in the performance of T0 + T1 MRI vs. T0 MRI alone. Typically, T0 + T1 MRI showed higher sensitivity compared to T0 MRI, meaning it was more effective in identifying pCR patients, especially in cases where tumors had a strong response to neoadjuvant chemotherapy. However, this also resulted in a slight decrease in specificity, suggesting that T0 + T1 MRI may lead to more false positives when assessing non-pCR cases. Notably, with the assistance of the STI model, radiologists improved their diagnostic performance. Compared with their independent assessments, AI-assisted radiologists generally exhibited higher sensitivity, while specificity remained largely stable, with only slight decreases observed in a few readers. These findings suggest that human-AI collaboration enhances diagnostic accuracy. To further explore the complementary value of human–AI collaboration, we also conducted a qualitative error analysis using representative cases, highlighting patterns of agreement and discrepancy between the STI model and radiologists. Detailed case-level interpretations and visual examples are provided in Supplementary A7 and Supplementary Figs. S9–S14.

### Prospective validation for STI

To assess the real-world clinical applicability of the STI model, we conducted a prospective validation study comparing its predictive performance against radiologists in determining pCR status. As shown in Fig. 5b, compared to radiologists, the STI model more accurately identified patients who achieved pCR, reducing the misclassification of pCR patients from 42.1% (8/19) to 17.4% (4/23), a decrease in error rate by 58.7%. Similarly, the misclassification rate for non-pCR patients was reduced from 26.7% (12/45) to 9.8% (4/41), with an error rate reduction of 63.3%. The confusion matrices in Fig. 5b further demonstrate that compared to radiologists, the model exhibited higher sensitivity in identifying pCR cases and better specificity in distinguishing non-pCR patients, indicating its potential to minimize overtreatment and undertreatment.

### STI model connected with survival

To assess the prognostic significance of the STI model’s NAC response predictions, we conducted survival analyses on RFS and OS. Kaplan–Meier survival curves demonstrated significant differences between patients classified into high STI score and low STI score groups based on their predicted STI scores (Fig. 5c). As expected, patients predicted to low risk exhibited superior survival outcomes compared to high risk patients, with statistically significant log-rank p-values observed for both RFS (p = 0.015) and OS (p = 0.0062). Furthermore, univariate Cox regression analysis (Supplementary Fig. S15) identified the STI model’s predicted response as a significant prognostic factor for both RFS and OS. In the RFS analysis, baseline tumor size and the deep learning-generated response score were found to be significant predictors, with a hazard ratio of 1.40 (95% CI: 1.03–1.88, p = 0.029) for baseline tumor size and 0.72 (95% CI: 0.53–1.00, p = 0.049) for the deep learning score. In the OS analysis, only the deep learning score was found to be a significant predictor, with a hazard ratio of 0.68 (95% CI: 0.46–1.00, p = 0.048), indicating that patients with a predicted favorable response had a lower risk of mortality. Other clinical variables, including molecular subtype, ER, PR, and HER2 status, did not show significant prognostic associations. These findings suggest that the STI model-generated response not only serves as a predictor of pCR but also holds strong prognostic value for long-term survival outcomes in breast cancer patients receiving NAC.

### Pathway enrichment and differential gene analysis

**Fig. 6:**
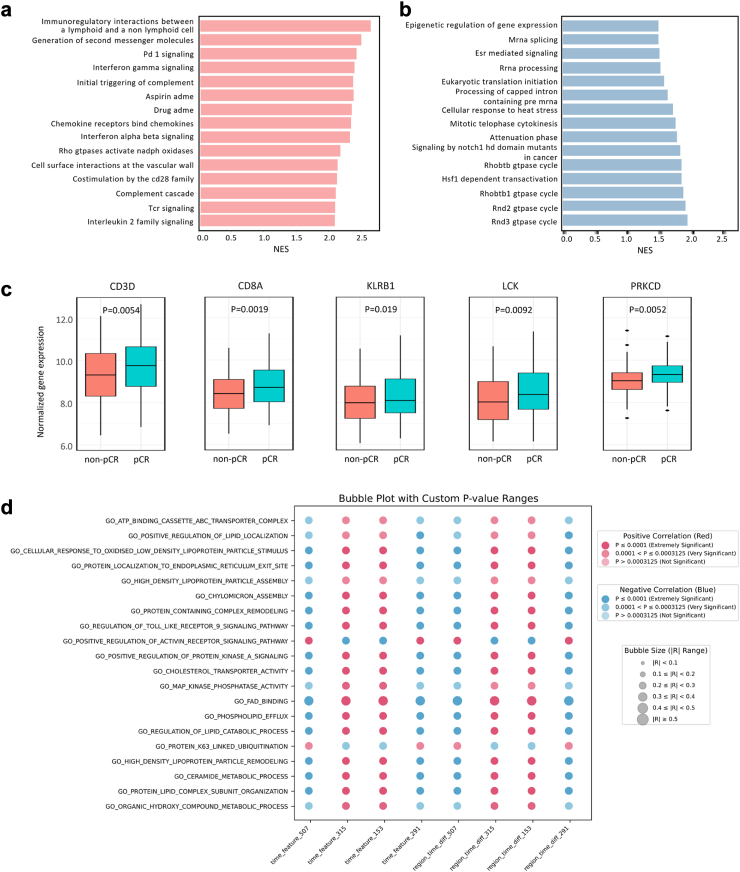
**Enriched pathways, gene expression, and tumor biological process correlations in STI model-predicted NAC response.** (a, b) GSEA using the C2 (curated gene sets) database identified significantly upregulated (a) and downregulated (b) pathways between different NAC response groups predicted by the STI model. (c) Box plots displaying the expression levels of key genes in different NAC response groups. (d) Bubble plot illustrating the correlation between deep features and tumor biological processes. Spearman correlation was used for association analysis. Bonferroni correction was applied to account for multiple testing across 160 comparisons (20 imaging features × 8 pathways). Based on this, we introduced two significance categories: (1) p ≤ 0.0001: highly significant; (2) p ≤ 0.0003125: statistically significant after Bonferroni correction. Note: GSEA = Gene Set Enrichment Analysis; STI = Spatiotemporal interaction model.

To explore the biological basis of NAC response predicted by the STI model, we conducted differential gene expression and pathway enrichment analyses. GSEA revealed significant upregulation of immune-related pathways in STI model-predicted pCR tumors, including PD-1 signaling, interferon-gamma signaling, and complement activation (Fig. 6a). In contrast, pathways linked to tumor progression and resistance, such as epigenetic regulation and RNA processing, were downregulated (Fig. 6b).

Further analysis of differentially expressed genes (DEGs) highlighted that T-cell activation markers, including CD3D (p = 0.0054) and CD8A (p = 0.0019), were significantly overexpressed in pCR tumors, suggesting a stronger immune response in tumors more likely to respond to NAC (Fig. 6c). Additionally, LCK (p = 0.0092), a key regulator of T-cell receptor signaling, was also elevated, reinforcing the role of an active immune microenvironment in treatment response. Correlation analysis (Fig. 6d) further confirmed that deep learning-extracted features were strongly associated with immune-related pathways, emphasizing that the STI model effectively captures tumor-immune interactions that influence chemotherapy outcomes.

### Immune microenvironment and pathological features

**Fig. 7:**
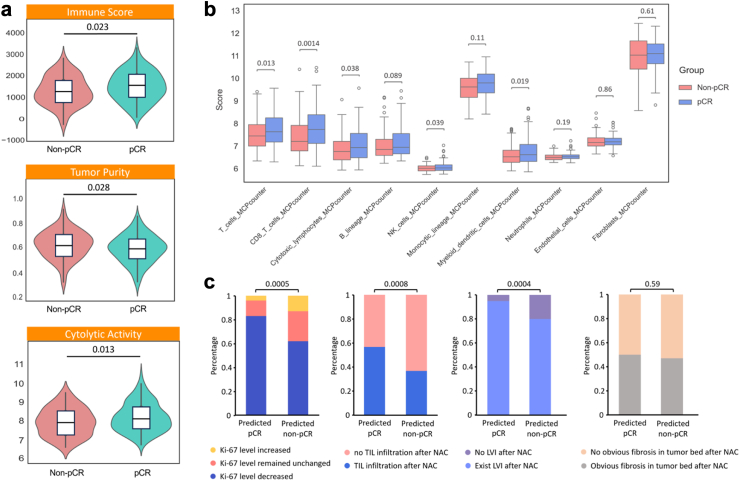
**Immune microphenotypes, and pathological characteristics in STI model-predicted NAC response groups.** (a) Immune characteristics and cytolytic activity between STI model-predicted groups (ESTIMATE and CTY scores). (b) Differences in immune cell abundance within the tumor microenvironment between STI model-predicted groups (MCPcounter). The Mann–Whitney U test was used to assess differences between the predicted pCR and non-pCR groups in (a) and (b). (c) Bar plots showing the distribution of pathological characteristics between predicted pCR and non-pCR groups, including Ki-67 level changes, TIL infiltration, LVI presence, and tumor bed fibrosis. The Chi-square test was used to evaluate differences in categorical pathological features between groups in (c). Note: CYT = cytolytic activity; TIL = tumor-infiltrating lymphocytes; LVI = lymphovascular invasion.

To further investigate the role of TME in NAC response, we analyzed immune infiltration characteristics and key pathological features predicted by the STI model (Fig. 7). Tumors predicted to achieve pCR exhibited significantly higher immune scores and cytolytic activity, along with lower tumor purity compared to non-pCR tumors (p < 0.05), indicating a more immune-active TME in pCR cases (Fig. 7a).

A detailed immune cell composition analysis (Fig. 7b) revealed that tumors predicted as pCR had significantly higher infiltration of CD8+ T cells, cytotoxic lymphocytes, and B cells, all of which are key mediators of antitumor immunity. In contrast, non-pCR tumors showed an enrichment of monocytic lineage cells and fibroblasts, which are often associated with immunosuppressive TMEs and poor chemotherapy response. These findings further support that the STI model captures immune-related features that influence NAC sensitivity.

Furthermore, we examined key pathological markers associated with tumor immunity, including Ki-67 expression, TILs, LVI, and tumor bed fibrosis (Fig. 7c). Compared to non-pCR cases, pCR-predicted patients exhibited a significant post-NAC decrease in Ki-67 expression (p = 0.0005) and increased TIL infiltration (p = 0.0008), reinforcing the association between strong adaptive immune responses and enhanced chemotherapy sensitivity. However, no significant differences in tumor bed fibrosis were observed between pCR and non-pCR groups (p = 0.59), implying that stromal remodeling may not be a primary factor in NAC outcomes.

## Discussion

In this study, we developed and validated the STI model for early predicting responses to NAC in breast cancer. By integrating spatial heterogeneity and temporal dynamics from longitudinal MRI data, the STI model significantly improved the accuracy of predicting NAC responses in breast cancer. Our research also validated the generalization capability of the STI model across multi-center datasets, demonstrating its stable and reliable performance across different cohorts, achieving AUC values ranging from 0.892 to 0.981. Compared to traditional clinical models and radiologists’ assessments, the STI model exhibited superior predictive performance across multiple cohorts. Furthermore, the biological interpretability analysis of the STI model revealed its relationship with immune-related pathways in the tumor microenvironment, providing crucial biological insights into the mechanisms of tumor treatment response.

In recent years, tumor spatial heterogeneity has become a significant area of research in cancer treatment and prognosis prediction.^37^ Traditional tumor analysis methods often treat tumors as homogeneous entities, neglecting the complexity of intratumoral heterogeneity. However, tumor spatial heterogeneity, including the cellular composition, blood vessel structures, and immune microenvironment of different regions within a tumor, directly influences tumor growth patterns, metastatic potential, and treatment responses.^38^ Therefore, capturing tumor spatial heterogeneity is crucial for accurately predicting cancer treatment responses and prognosis. Many studies have demonstrated that integrating information from different regions within the tumor significantly improves the accuracy of tumor prognosis prediction.^23^, ^24^, ^25^ In our model, the spatial information of the tumor is derived not only from its overall morphology but also from the feature extraction of different subregions within the tumor. Based on the work of Li et al.,^25^ we refined the spatial heterogeneity within the tumor into multiple spatial units, thereby providing more accurate tumor region features for subsequent prediction, and enhancing the model’s ability to perceive changes in the local tumor microenvironment. Experimental results show that this architecture improves the prediction AUC of NAC response by 13.4% in the external validation cohort compared to global feature methods.

Beyond spatial features, tumor time dynamics also play a crucial role in predicting treatment responses.^39^^,^^40^ Traditional NAC response prediction methods primarily rely on pre-treatment imaging data, which fail to fully reflect the dynamic changes in the tumor over the course of treatment. By integrating multiple time-point data, our STI model combines tumor time dynamics, capturing changes over time to enhance the model’s performance. Previous studies have also demonstrated that using multiple time points of imaging significantly improves prediction accuracy compared to single time-point models. For example, Zeng et al.^27^ utilized DCE-MRI to predict the response to neoadjuvant therapy, demonstrating promising performance with the use of delta radiomics. Similarly, Huang et al.^30^ developed a stacking model based on longitudinal multi-parametric MRI, achieving high diagnostic accuracy. These studies underscore the advantages of incorporating multi-time-point imaging data, as opposed to single time-point models, which often provide limited predictive capability.

Based on previous research, our STI model incorporates a multi-head attention mechanism to integrate spatial and temporal information. This mechanism dynamically aggregates features from different time points through weighted attention, enabling the model to effectively capture tumor dynamics and accurately reflect the complex evolution of the tumor throughout the treatment process. Compared to other longitudinal data-based models, the STI model successfully integrates spatiotemporal features, resulting in a 65.1% and 13.5% improvement in AUC for predicting NAC response in the external validation cohort compared to the T0 + Spatial and T1 + Spatial models, respectively. In addition to the improvements in AUC, our model demonstrates strong NPV (0.921 [0.872–0.956] in the EVC total), which is crucial for accurately identifying non-pCR patients and adjusting treatment plans accordingly. While the PPV is moderate due to the imbalanced nature of the dataset, similar studies have reported comparable PPV values for pCR prediction, highlighting the challenges in predicting low-incidence events like pCR.^31^ Nonetheless, the high NPV reinforces the model’s clinical value in identifying non-pCR patients early, thereby optimizing treatment strategies and improving patient outcomes. Through ablation studies, we further validated the impact of the spatiotemporal attention mechanism on model performance. The results indicate that the spatiotemporal interaction mechanism significantly enhances the model’s predictive power. Furthermore, the model maintains robust predictive performance across different clinical stages and molecular subtypes, highlighting its versatility and wide applicability.

To further assess the superiority of our model over clinical models and radiologist evaluations, we conducted a comparative analysis. The STI model significantly outperformed the conventional clinical model, which incorporated pathological factors such as molecular subtype and receptor status, demonstrating a notably higher predictive capability.^29^ Surprisingly, the integration of clinical predictors into the STI model did not enhance its predictive performance. These findings suggest that the STI model alone serves as a highly effective predictor of NAC response, with clinical variables providing no substantial improvement in predictive accuracy. Moreover, the STI model exhibited superior performance compared to experienced radiologists in predicting pCR, further reinforcing its potential as an AI-assisted diagnostic tool.^31^ Notably, with the assistance of the STI model, radiologists’ diagnostic performance improved significantly. Compared to their independent evaluations, AI-assisted radiologists generally exhibited higher sensitivity, while specificity remained largely stable. This highlights the model’s ability to support radiologists in more accurately identifying pCR patients. Thus, the collaboration between human expertise and AI enhances diagnostic accuracy, making the STI model a promising tool to aid clinical decision-making, particularly in evaluating early treatment responses.

Recognizing the need for real-world validation, we conducted a prospective evaluation of the STI model, confirming its robustness in a clinical setting. The STI model outperformed radiologists’ assessments, with a 58.7% reduction in error rate for predicting pCR, achieving an accuracy of 82.6% (19/23), and a 63.3% reduction in error rate for predicting non-pCR, with an accuracy of 90.2% (37/41). For non-pCR patients, early prediction enables adjustments to the treatment plan to reduce toxicity and improve treatment efficiency. For pCR patients, maintaining the current treatment could yield benefits, potentially allowing for less invasive interventions, such as breast-conserving surgery and the omission of axillary lymph node dissection. These findings emphasize the clinical value of the STI model in guiding personalized treatment strategies for breast cancer patients undergoing NAC.

Beyond immediate treatment guidance, we investigated the prognostic value of STI model predictions. Our survival analysis revealed that patients predicted to have a high STI score exhibited significantly improved RFS and OS compared to those predicted to have a low STI score. These findings align with those of Fan et al.,^41^ who demonstrated that NAC response patterns were strongly correlated with survival outcomes. Furthermore, univariate Cox regression analysis indicated that the STI model score remained an independent prognostic factor, highlighting its clinical utility beyond NAC response prediction.

Deep learning models are often criticized for their “black-box” nature, making interpretability a critical factor for clinical adoption. To address this, we investigated the biological basis of the STI model’s predictions, focusing on its association with the tumor immune microenvironment. Our analysis revealed that tumors predicted to achieve pCR exhibited distinct immune characteristics, including increased expression of immune-related genes (e.g., CD3D, CD8A, and LCK) and significant enrichment of immune signaling pathways such as PD-1 signaling, interferon-gamma signaling, and complement activation. These findings suggest that the STI model effectively captures immune activation patterns that are linked to enhanced NAC responses.

Moreover, immune microenvironment characterization demonstrated that STI-predicted pCR tumors were significantly associated with increased CD8+ T cell and cytotoxic lymphocyte infiltration, lower tumor purity, and higher cytolytic activity, reinforcing the model’s ability to reflect key aspects of TME composition. This is consistent with prior study.^42^^,^^43^ Mao et al.^43^ employed a deep learning model to predict axillary pCR and found that high model scores were associated with the upregulation of immune-related genes and increased infiltration of monocytes and macrophages. Similarly, Huang et al.^42^ reported elevated immune activity in tumors with favorable NAC responses. Together, these findings support the immunological basis of deep learning–based NAC response prediction. In addition to immune signatures, pathological analysis confirmed significant associations between STI-predicted responses and key histological features, including post-NAC Ki-67 reduction, increased TILs, and reduced LVI. These results further emphasize the model’s ability to capture tumor biology beyond imaging, providing insights into how chemotherapy response is mediated through both immune dynamics and proliferative changes. Taken together, these results demonstrate that the STI model effectively captures the immune landscape of breast tumors and its association with NAC response. The strong correlation between model predictions and immune-related features highlights the potential of integrating spatiotemporal tumor dynamics for precision oncology and treatment stratification.

Despite its promising performance, our study has several limitations. First, although we included a prospective validation cohort, selection bias is inevitable in a retrospective study design. The generalizability of our findings to broader populations remains uncertain, necessitating further validation through international clinical trials. Second, while DCE-MRI was used as the primary imaging modality-specifically peak-enhancement phase images, additional MRI sequences (e.g., T2-weighted imaging and diffusion-weighted imaging) could provide complementary information and could potentially improve model performance. Future studies incorporating multi-parametric MRI data are warranted. Third, our study primarily focuses on breast tumors. Expanding the model to incorporate simultaneous axillary lymph node involvement would further enhance its clinical applicability. Fourth, while we conducted transcriptomic analyses to support model interpretability, these findings were based on publicly available databases rather than hospital-based prospective sequencing data. Future studies should incorporate large-scale, single-cell RNA sequencing to gain deeper biological insights. Fifth, although external and prospective validation cohorts were included, their sample sizes were relatively small, which may limit the statistical power and generalizability of the results. Further expansion of sample size and inclusion of additional centers are warranted to enhance the robustness and clinical applicability of the STI model.

In summary, the STI model demonstrated excellent performance as a noninvasive tool for predicting NAC response in breast cancer patients. By integrating spatial–temporal interactions, the model effectively captured tumor evolution and outperformed conventional clinical models and radiologists’ assessments. In addition, our study incorporated gene expression data to provide biological interpretability, reinforcing the model’s ability to reflect tumor characteristics at both the imaging and molecular levels. These findings highlight the advantage of integrating spatial and temporal features within a deep learning framework, enabling the STI model to capture complex tumor interactions over time. This suggests its potential as a valuable tool for personalized treatment planning. Future studies may also explore incorporating multi-omics data, such as proteomics or metabolomics, to further enrich the model’s predictive capacity.

## Contributors

Guarantors of integrity of entire study, Q.W. Z., N. M., Y. G.; study concepts/study design or data acquisition or data analysis/interpretation, all authors; manuscript drafting or manuscript revision for important intellectual content, all authors; approval of final version of submitted manuscript, all authors; agrees to ensure any questions related to the work are appropriately resolved, all authors; literature research, W.J. T., Q.C. K., clinical studies, W.J. T., G.Y., S.S. D., B.H. L., Y. L., M.N.; experimental studies, W.J. T., C.J., Q.W. Z., S.Y. C., Y.X. C., Y.G.; statistical analysis, W.J. T., C. J., ; and manuscript editing, W.J. T., C.J., Q.W. Z., Y.G. W.J.T. and C.J. have verified the underlying data. All authors read and approved the final version of the manuscript, and ensure it is the case.

## Data sharing statement

As the study involved human participants, the data cannot be made freely available in the manuscript or in a public repository because of ethical restrictions. However, the data are available from the Guangzhou First People’s Hospital to researchers who meet the criteria for access to confidential data. Interested researchers can send data access requests to the corresponding author (Y. G.). The source code of the deep learning network in this study is available at https://github.com/JBnoJB/STIModel.

## Declaration of interests

All authors declare no competing interests.

## References

[1] Bray F., Laversanne M., Sung H. (2024). Global cancer statistics 2022: GLOBOCAN estimates of incidence and mortality worldwide for 36 cancers in 185 countries. CA Cancer J Clin.

[2] Korde L.A., Somerfield M.R., Carey L.A. (2021). Neoadjuvant chemotherapy, endocrine therapy, and targeted therapy for breast cancer: ASCO guideline. J Clin Oncol.

[3] Gralow J.R., Burstein H.J., Wood W. (2008). Preoperative therapy in invasive breast cancer: pathologic assessment and systemic therapy issues in operable disease. J Clin Oncol.

[4] Cortazar P., Zhang L., Untch M. (2014). Pathological complete response and long-term clinical benefit in breast cancer: the CTNeoBC pooled analysis. Lancet.

[5] van Mackelenbergh M.T., Loibl S., Untch M. (2023). Pathologic complete response and individual patient prognosis after neoadjuvant chemotherapy plus anti-human epidermal growth factor receptor 2 therapy of human epidermal growth factor receptor 2-positive early breast cancer. J Clin Oncol.

[6] Fayanju O.M., Ren Y., Thomas S.M. (2018). The clinical significance of breast-only and node-only pathologic complete response (pCR) after neoadjuvant chemotherapy (NACT): a review of 20,000 breast cancer patients in the national cancer data base (NCDB). Ann Surg.

[7] Haque W., Verma V., Hatch S., Suzanne Klimberg V., Brian Butler E., Teh B.S. (2018). Response rates and pathologic complete response by breast cancer molecular subtype following neoadjuvant chemotherapy. Breast Cancer Res Treat.

[8] Romeo V., Accardo G., Perillo T. (2021). Assessment and prediction of response to neoadjuvant chemotherapy in breast cancer: a comparison of imaging modalities and future perspectives. Cancers (Basel).

[9] Wang H., Mao X. (2020). Evaluation of the efficacy of neoadjuvant chemotherapy for breast cancer. Drug Des Devel Ther.

[10] Fowler A.M., Mankoff D.A., Joe B.N. (2017). Imaging neoadjuvant therapy response in breast cancer. Radiology.

[11] Mann R.M., Kuhl C.K., Kinkel K., Boetes C. (2008). Breast MRI: guidelines from the European society of breast imaging. Eur Radiol.

[12] (2024). NCCN guidelines: breast cancer, Version 5.

[13] Rauch G.M., Adrada B.E., Kuerer H.M., van la Parra R.F., Leung J.W., Yang W.T. (2017). Multimodality imaging for evaluating response to neoadjuvant chemotherapy in breast cancer. AJR Am J Roentgenol.

[14] Hylton N.M., Blume J.D., Bernreuter W.K. (2012). Locally advanced breast cancer: MR imaging for prediction of response to neoadjuvant chemotherapy--results from ACRIN 6657/I-SPY TRIAL. Radiology.

[15] Schwartz L.H., Seymour L., Litiere S. (2016). Recist 1.1 - standardisation and disease-specific adaptations: perspectives from the RECIST working group. Eur J Cancer.

[16] Lambin P., Rios-Velazquez E., Leijenaar R. (2012). Radiomics: extracting more information from medical images using advanced feature analysis. Eur J Cancer.

[17] Bi W.L., Hosny A., Schabath M.B. (2019). Artificial intelligence in cancer imaging: clinical challenges and applications. CA Cancer J Clin.

[18] Vasan N., Baselga J., Hyman D.M. (2019). A view on drug resistance in cancer. Nature.

[19] Eisenstein M. (2024). Why tumour geography matters - and how to map it. Nature.

[20] Zhang X., Su G.H., Chen Y., Gu Y.J., You C. (2023). Decoding intratumoral heterogeneity: clinical potential of habitat imaging based on radiomics. Radiology.

[21] Gillies R.J., Balagurunathan Y. (2018). Perfusion MR imaging of breast cancer: insights using “habitat imaging”. Radiology.

[22] Xie P., Huang Q., Zheng L. (2024). Sub-region based histogram analysis of amide proton transfer-weighted MRI for predicting tumor budding grade in rectal adenocarcinoma: a prospective study. Eur Radiol.

[23] Wu J., Cao G., Sun X. (2018). Intratumoral spatial heterogeneity at perfusion MR imaging predicts recurrence-free survival in locally advanced breast cancer treated with neoadjuvant chemotherapy. Radiology.

[24] Kim J.Y., Kim J.J., Hwangbo L. (2020). Kinetic heterogeneity of breast cancer determined using computer-aided diagnosis of preoperative MRI scans: relationship to distant metastasis-free survival. Radiology.

[25] Shi Z., Huang X., Cheng Z. (2023). MRI-Based quantification of intratumoral heterogeneity for predicting treatment response to neoadjuvant chemotherapy in breast cancer. Radiology.

[26] Fan M., Chen H., You C. (2021). Radiomics of tumor heterogeneity in longitudinal dynamic contrast-enhanced magnetic resonance imaging for predicting response to neoadjuvant chemotherapy in breast cancer. Front Mol Biosci.

[27] Zeng Q., Xiong F., Liu L., Zhong L., Cai F., Zeng X. (2023). Radiomics based on DCE-MRI for predicting response to neoadjuvant therapy in breast cancer. Acad Radiol.

[28] Zeng Q., Ke M., Zhong L. (2023). Radiomics based on dynamic contrast-enhanced MRI to early predict pathologic complete response in breast cancer patients treated with neoadjuvant therapy. Acad Radiol.

[29] Liu Y., Wang Y., Wang Y. (2022). Early prediction of treatment response to neoadjuvant chemotherapy based on longitudinal ultrasound images of HER2-positive breast cancer patients by Siamese multi-task network: a multicentre, retrospective cohort study. eClinicalMedicine.

[30] Huang Y., Zhu T., Zhang X. (2023). Longitudinal MRI-based fusion novel model predicts pathological complete response in breast cancer treated with neoadjuvant chemotherapy: a multicenter, retrospective study. eClinicalMedicine.

[31] Gao Y., Ventura-Diaz S., Wang X. (2024). An explainable longitudinal multi-modal fusion model for predicting neoadjuvant therapy response in women with breast cancer. Nat Commun.

[32] Allison K.H., Hammond M.E.H., Dowsett M. (2020). Estrogen and progesterone receptor testing in breast cancer: American society of clinical oncology/college of American pathologists guideline update. Arch Pathol Lab Med.

[33] Loibl S., Gianni L. (2017). HER2-positive breast cancer. Lancet.

[34] Cortazar P., Geyer C.E. (2015). Pathological complete response in neoadjuvant treatment of breast cancer. Ann Surg Oncol.

[35] Esserman L.J., Berry D.A., DeMichele A. (2012). Pathologic complete response predicts recurrence-free survival more effectively by cancer subset: results from the I-SPY 1 TRIAL--CALGB 150007/150012, ACRIN 6657. J Clin Oncol.

[36] Zhang J., Cui Z., Shi Z. (2023). A robust and efficient AI assistant for breast tumor segmentation from DCE-MRI via a spatial-temporal framework. Patterns (N Y).

[37] Zhao S., Chen D.P., Fu T. (2023). Single-cell morphological and topological atlas reveals the ecosystem diversity of human breast cancer. Nat Commun.

[38] Zhao N., Rosen J.M. (2022). Breast cancer heterogeneity through the lens of single-cell analysis and spatial pathologies. Semin Cancer Biol.

[39] de Visser K.E., Joyce J.A. (2023). The evolving tumor microenvironment: from cancer initiation to metastatic outgrowth. Cancer Cell.

[40] Bollen Y., Stelloo E., van Leenen P. (2021). Reconstructing single-cell karyotype alterations in colorectal cancer identifies punctuated and gradual diversification patterns. Nat Genet.

[41] Fan M., Wang K., Pan D. (2024). Radiomic analysis reveals diverse prognostic and molecular insights into the response of breast cancer to neoadjuvant chemotherapy: a multicohort study. J Transl Med.

[42] Huang Y.H., Shi Z.Y., Zhu T. (2025). Longitudinal MRI-driven multi-modality approach for predicting pathological complete response and B cell infiltration in breast cancer. Adv Sci.

[43] Li Z., Gao J., Zhou H. (2024). Multiregional dynamic contrast-enhanced MRI-based integrated system for predicting pathological complete response of axillary lymph node to neoadjuvant chemotherapy in breast cancer: multicentre study. EBioMedicine.

