# Supplementary material for: Development and validation of an MRI spatiotemporal interaction model for early noninvasive prediction of neoadjuvant chemotherapy response in breast cancer: a multicentre study
Source: eClinicalMedicine. 2025 Jun 12;85:103298. doi: 10.1016/j.eclinm.2025.103298 (PMC12205660; doi:10.1016/j.eclinm.2025.103298)
Supplement: Supplementary Figs. S1–S15 and Tables S1–S11 [file mmc1.docx]

**Supplementary information for**

**MRI Spatiotemporal Interaction Model for Early Noninvasive Predicting Neoadjuvant Chemotherapy Response in Breast Cancer: multicentre study**

Supplementary Appendix 1: Patient enrollment criteria and sample size estimation

Supplementary Appendix 2: Image protocols and images preprocessing

Supplementary Appendix 3: Breast tumor 3D segmentation model

Supplementary Appendix 4: Deep learning network design and training

Supplementary Appendix 5: Development of the STI model integrated with clinical characteristics

Supplementary Appendix 6: Ablation analysis for STI

Supplementary Appendix 7: Reader study error analysis

**Supplementary Appendix 1. Patient enrollment criteria and sample size estimation**

**Patient enrollment criteria**

This multicenter study retrospectively and prospectively collected breast cancer patients who received NAC from multiple institutions and public databases. The study included four cohorts: (i) a primary cohort for model development and internal validation, (ii) an external validation cohort from independent hospitals, (iii) a prospective cohort for clinical feasibility assessment, and (iv) a public database cohort for prognostic and biological interpretability validation.

The primary cohort initially included 483 patients from two hospitals. These patients were selected based on the availability of longitudinal MRI scans (pre-NAC and early-NAC) and complete clinical and pathological information. Exclusion criteria included: (1) Missing MRI data before (T0) and/or after NAC (T1) (T0: Pre-NAC, refers to MRI examinations performed within 2 weeks prior to the start of NAC; T1: early-NAC, refers to MRI examinations performed within the completion of 2 cycles of NAC); (2) Poor MRI quality or missing DCE-MRI images; (3) Absence of post-NAC surgical outcomes, a total of 323 patients were included in the primary cohort (GZ: n = 176, LZ: n = 147). To evaluate the generalizability of the model, an external validation cohort comprised 599 breast cancer participants across three independent hospitals. Similar exclusion criteria were applied, 309 patients were retained from three independent hospitals: DG dataset (EVC1, n = 95); YT dataset (EVC2, n = 131); YN dataset (EVC3, n = 83). A prospective cohort was collected from the GZ hospital to further assess the real-world clinical feasibility of the STI model. After excluding those with missing MRI data before and/or after NAC (n = 9) and those without surgical outcomes at the time of study (n = 5), 64 patients enrolled in the prospective cohort.

The public database cohort initially consisted of 606 patients from two datasets (ACRIN 6657/I-SPY1: n = 221, ACRIN 6698/I-SPY2: n = 385). The exclusion criteria included missing MRI data before and/or after NAC (absence of MRI examinations at T0 and/or T1 time points), absence of surgical outcomes or missing pathological data, and poor MRI image quality or missing DCE-MRI images. After applying these exclusions, 126 patients from ACRIN 6657/I-SPY1 were included for prognostic validation, and 222 patients from ACRIN 6698/I-SPY2 were included for biological interpretability analysis. For the ACRIN 6657 dataset, patients were re-scanned approximately 21 days after the first cycle of anthracycline-based treatment (T1). For the ACRIN 6698 dataset, the re-scan took place 3 weeks after the initial treatment (T1).

**Sample size estimation:**

The sample size calculation was performed using MedCalc Statistical Software (MedCalc Software Ltd, version 23.2.1), specifically the "Sample size: area under ROC curve" module. The calculation was based on the following assumptions: an expected area under the ROC curve (AUC) of 0.80 under the alternative hypothesis, compared to a null hypothesis value of 0.50; a two-sided significance level (α) of 0.05; a power of 90% (1−β = 0.90); and a sample size allocation ratio of 7:3 between the negative and positive groups. Under these parameters, the minimum required sample size was determined to be 44 patients, including 13 positive cases and 31 negative cases.

In this study, the primary cohort (PC) included 323 patients (pCR: 84, non-pCR: 239); the three external validation cohorts included 95 patients (pCR: 35, non-pCR: 60), 131 patients (pCR: 38, non-pCR: 93), and 83 patients (pCR: 26, non-pCR: 57), respectively—all of which exceeded the minimum sample size requirement.

**Supplementary Appendix 2: Image protocols and** **images preprocessing**

**Image protocols**

For the main cohort, all patients in the GZ dataset underwent imaging using a 1.5T MRI system (uMR 560, United Imaging) with a dedicated 4-channel SENSE breast coil. The standard DCE-MRI protocol began with pre-enhancement T1-weighted images, followed by the intravenous injection of gadolinium chelate contrast agent (GDDTPA, 0.1 mmol/kg). In the LZ dataset, imaging was conducted with a Philips 3.0T MRI system (Achieva) with its corresponding DCE protocol. For the prospective cohort, the MRI parameters from the GZ dataset were applied, as previously described.

For the external validation cohort, the DG dataset used a Philips 3.0T MRI system (Ingenia), while the YT dataset used a GE 3.0T MRI system (Discovery MR750W). Both systems followed their respective configurations for standard DCE protocols. The YN dataset, like the DG dataset, used a Philips 3.0T MRI system (Ingenia) with its corresponding DCE protocol. For further details on MRI acquisition protocols, please refer to Table S1.

For the ACRIN 6657/I-SPY1 cohort, the DCE-MRI protocol was described in previous study[1]. In brief, the imaging was performed using a 1.5T breast DCE-MRI with a dedicated breast radiofrequency coil. The TR/TE were ≤20/4.5 ms; the flip angle was ≤45°; the FOV was 16-18 cm; the minimum matrix was 256 × 192; and there were 64 sections with a thickness of 2.5 mm or less.

For the ACRIN 6698/I-SPY2 cohort, the DCE-MRI protocol was also described elsewhere[2], with a 1.5- or 3.0-T field strength magnet and a dedicated breast radiofrequency coil. The TR of greater than 4000 msec; echo time minimum; the flip angle was 90°; the FOV was 30–36 cm; acquired matrix, 128 × 128 to 192 × 192; section thickness was 4–5 mm.

To ensure consistency across centers with varying DCE-MRI protocols, we individually selected the post-contrast phase corresponding to peak tumor enhancement for each patient. Peak enhancement was defined as the phase exhibiting the highest average signal intensity within the tumor region, typically occurring 60-120 seconds after contrast injection, in accordance with the ACR BI-RADS Atlas (5th edition) and the 2023 BI-RADS update (Fig. S2)[3]. This individualized approach allowed for consistent identification of the peak enhancement phase across heterogeneous imaging protocols.

**Images preprocessing**

The phase with peak tumor enhancement at DCE-MRI was used to preprocess. Image preprocessing includes N4 bias field correction[4], resampling using nearest-neighbor interpolation for binary data or linear interpolation for non-binary data, and histogram normalization of the image intensity values[5]. To meet the dimensional requirements of subsequent image processing methods, affine transformation combined with mutual information-based metrics is used for image registration. Additionally, to accommodate two different use cases, we perform registration on different target regions: one based on full-image registration and the other based on rigid registration of a tumor region. Due to variations in image acquisition devices and protocols across multiple centers, —especially variations between 1.5T and 3T scanners—we implemented an additional harmonization step to standardize image intensity distributions. Inspired by Fourier Domain Adaptation (FDA), we applied a Fourier-based transformation to align the low-frequency amplitude spectrum of input images to a reference domain [6]. This approach reduces scanner-related variability in contrast and brightness while preserving high-frequency structural information, thereby improving model robustness across centers.

**Supplementary Appendix 3: Breast tumor 3D segmentation model**

In this study, we developed a 3D spatial-temporal network for breast tumor segmentation using the PyTorch framework[7]. The model takes as input both raw DCE-MRI images and subtraction images, aiming to capture dynamic changes in tumors across different phases. The model employs a U-Net architecture with residual blocks, consisting of 4 downsampling and upsampling modules, and utilizes skip connections to preserve detailed information. The Spatial-Temporal Transformer consists of both spatial and temporal transformers, which model spatial features within a single phase and dynamic changes across different phases, respectively. Through the attention mechanism, the model automatically handles missing phases, enhancing its robustness. The training process uses the Adam optimizer, with an initial learning rate of 0.002 for the full breast segmentation model and 0.001 for the tumor segmentation model. The learning rate is halved every 50 epochs, and the model is trained for a total of 300 epochs with a batch size of 2. The training process includes over 200,000 iterations to optimize resource usage and improve learning efficiency. The model's code is publicly available on GitHub. Subsequently, two experienced radiologists used ITK-SNAP software (version 4.0.1, [http://www.itksnap.org/](http://www.itksnap.org/)) to review the automatically segmented tumor ROIs from multi-center data and resolved any discrepancies through consensus. The Dice Similarity Coefficient (DSC) was calculated to assess the similarity between the model's automatic segmentation and the ROIs adjusted by the radiologists.

The 3D spatial-temporal network demonstrates satisfactory segmentation performance, with the average DSC for tumors ranging from 0.84 to 0.92 across all datasets. The results of the automated segmentation are shown in Supplementary Figure S2.

**Supplementary Appendix 4: Deep learning network design and training**

In this study, we propose a non-invasive STI mode based on MRI for early prediction of NAC response in breast cancer patients. The model development consists of several key components: (1) Tumor habitat generation; (2) Pretraining; (3) Model architecture and spatiotemporal feature integration, all of which contribute to the unique integration of spatial and temporal features from MRI images; (4) Model output and prediction.

(1) Tumor habitat generation

After segmenting the whole tumor region, intratumoral subregion segmentation was performed based on prior study[8]. Specifically, radiomics features (including first-order features, texture features, and gradient features) were extracted from the whole tumor regions using PyRadiomics[9]. Subsequently, this study applies the K-means method to further clustered habitats. Three to six cluster count ranges were evaluated. Select the optimal number of clusters according to the Bayesian information criterion (BIC) [10] and Calinski-Harabasz (CH) [11] scores.

The results suggest that the optimal number of clusters was determined to be 3, as it yielded the highest CH score and the lowest BIC score (Table S2, Fig. S4), indicating the best balance between intra-cluster compactness and model complexity.

(2) Pretraining

Before model training, we conducted large-scale contrastive learning pre-training with MedicalNet on the Duke-Breast-Cancer-MRI public breast MRI datasets (https://www.cancerimagingarchive.net/collection/duke-breast-cancer-mri/) to better initialize the model's parameters. This dataset consists of breast DCE-MRI scans, which are highly relevant to our task. The goal was to capture feature distributions through initial training on a large dataset, enhancing the model’s generalization ability.

(3) Model architecture and spatiotemporal feature integration

The segmented breast image was cropped to focus on the tumor and surrounding area. Files representing the optimal number of subregions are treated as spatial information and processing intervals are treated as time data. To enhance the model's predictive performance, we designed a Spatiotemporal Interactive Network using a Siamese network architecture. The input to the network includes images from two time points (T0 and T1) at the whole tumor level and subregion level. For each image, features were extracted and concatenated according to specified weights. Temporal information, such as the time interval between T0 and T1, was incorporated using a temporal attention mechanism. The attention mechanism was designed to learn the dynamic interactions between spatial and temporal features, allowing the model to capture subtle changes in the tumor's characteristics over time. The core of our model architecture involves a MultiHeadTemporalAttention layer, inspired by the Transformer architecture, which has shown success in various sequence modeling tasks, such as natural language processing. The key feature of this layer is its ability to integrate time-related information effectively with spatial features. In traditional attention mechanisms, each feature is compared with others to determine their relevance; however, the MultiHeadTemporalAttention layer extends this by incorporating explicit time encoding (TD). This allows the model to treat time intervals between scans as part of the attention computation, thus improving the model's understanding of the temporal dynamics of tumor progression.

Its main components include:

**Time Encoding Projection**: The time interval (TD) between T0 and T1 is first encoded using a neural network layer to project it into a feature space that aligns with the dimensionality of the input features. This enables the model to learn the impact of varying time intervals on the features and the prediction task, such as how the time gap between scans affects tumor response to treatment.

**Multi-Head Attention Mechanism**: The projected time encoding is combined with spatial feature representations to generate the query (Q), key (K), and value (V) vectors used in the attention mechanism. The multi-head structure splits the attention mechanism into multiple heads, allowing the model to capture different temporal relationships (e.g., short-term and long-term dependencies) across multiple feature subspaces. This parallel attention mechanism enhances the model's ability to process and understand complex time-dependent data.

**Attention Score Calculation**: The attention scores are computed using the scaled dot-product attention mechanism. These scores indicate how well the time-encoded features (K) align with the spatial features (Q). By normalizing the scores with a softmax function, the model generates attention weights, which are then used to aggregate the most relevant information from different time points.

**Residual Connections and Normalization**: To improve stability and facilitate learning, residual connections and Layer Normalization are employed. These techniques help mitigate the issues of vanishing or exploding gradients, which are common in deep learning models, and enhance the model’s convergence during training.

**Feedforward Network (FFN)**: After the attention mechanism, a feedforward network (FFN) is used to process the output features. The FFN consists of fully connected layers that perform additional non-linear transformations to capture higher-level abstractions of the temporal and spatial information.

(4) Model output and prediction

The output of the STI model is a continuous probability score indicating the likelihood of achieving pCR. For classification purposes, a probability threshold was selected based on the ROC curve analysis, using the point on the ROC curve closest to (0,1)—which represents the optimal trade-off between sensitivity and specificity.

**Supplementary Appendix 5: Development of the STI model integrated with clinical characteristics**

In this study, deep features were extracted from the final feature representation layer of the STI model, specifically, the 2048-dimensional vector output after the spatiotemporal attention module, but before the final classifier. A genetic algorithm (GA) was then applied to select the optimal subset of these deep features for integration into the model. The GA was configured with a population size of 100, 30 generations, a crossover probability of 0.7, and a mutation probability of 0.2. Tournament selection and uniform integer mutation were used during optimization. Importantly, clinical features (HER2, ER, PR, and molecular subtype) were fixed during the GA process, ensuring that these variables were included in the final feature subset for the integrated model. The remaining deep features were selected by the GA, which aimed to maximize the performance of the combined model. Model performance for each feature subset was evaluated using 5-fold cross-validation.

Simultaneously, clinical features were filtered through univariate and multivariate logistic regression analyses, retaining the four features (HER2, ER, PR, and molecular subtype) with statistical significance. The final integrated model comprised 20 features, combining 16 GA-selected deep features and the four fixed clinical variables. The Support Vector Machine (SVM) was employed as the classifier for both the STI and clinical models, maintaining consistency across all model types.

**Supplementary Appendix 6:** **Ablation analysis for STI**

To assess the necessity of different components in the proposed spatiotemporal interaction network for accurately predicting multicenter data responses, we conducted ablation experiments by modifying the network structure. First, we removed the spatial attention module while keeping the dimensionality of the vector input and the subsequent modules unchanged, and performed predictions. Then, we eliminated the temporal attention module and instead used a simple linear rate of change to calculate feature variations over time, without applying the Transformer-based temporal attention mechanism for subsequent predictions. Additionally, we performed an ablation experiment where we removed the preprocessing steps, including the Fourier-based domain adaptation and harmonization process. The model was trained and tested without these preprocessing steps to evaluate their impact on model performance. Lastly, we conducted an experiment where we removed the pretraining phase, and trained the model directly on the target dataset without the initial pretraining on publicly available breast MRI datasets. This allowed us to evaluate the impact of pretraining on the model’s ability to generalize and improve performance. In all ablation experiments, the models were trained using the same approach as the main spatiotemporal model to obtain the results. These experiments help identify the importance of each component and contribute to a better understanding of the model’s overall performance and generalizability.

**Supplementary Appendix 7: Reader study error analysis**

To further understand the predictive behavior of the STI model and its complementarity with radiologists, we performed a qualitative error analysis by reviewing representative cases from the reader study. We observed that in most cases, the STI model's predictions were closely aligned with those of radiologists (e.g., Supplementary Fig. S9 and S10), especially when the imaging features showed evident tumor shrinkage or no apparent change. This consistency highlights the STI model's alignment with expert judgment under typical imaging patterns.

However, discrepancies did arise. Certain challenging cases (e.g., Supplementary Fig. S11 and S12) were also often misclassified by radiologists, indicating intrinsic imaging ambiguity. In some instances, radiologists successfully identified these cases based on contextual clinical cues, such as HER2 positivity or low T stage, which may not be fully captured by the imaging features alone.

Notably, we also found that AI-assisted decision-making improved radiologist performance in several cases. For example, in cases where the initial radiologist prediction was incorrect, access to the STI model's output led to correct revisions in their assessment (e.g., Supplementary Fig. S13 and S14). These findings support the clinical value of the STI model not only as an independent decision support system but also as a tool to enhance human diagnostic accuracy.

**Reference**

1 Hylton NM, Blume JD, Bernreuter WK et al (2012) Locally advanced breast cancer: MR imaging for prediction of response to neoadjuvant chemotherapy--results from ACRIN 6657/I-SPY TRIAL. Radiology 263:663-672

2 Partridge SC, Zhang Z, Newitt DC et al (2018) Diffusion-weighted MRI Findings Predict Pathologic Response in Neoadjuvant Treatment of Breast Cancer: The ACRIN 6698 Multicenter Trial. Radiology 289:618-627

3 BI-RADS 6th Edition Updates and Challenging Cases (2024). RSNA Course Catalog

4 Tustison NJ, Avants BB, Cook PA et al (2010) N4ITK: improved N3 bias correction. IEEE Trans Med Imaging 29:1310-1320

5 Nyul LG, Udupa JK, Zhang X (2000) New variants of a method of MRI scale standardization. IEEE Trans Med Imaging 19:143-150

6 Yang Y, Soatto S (2020) FDA: Fourier Domain Adaptation for Semantic Segmentation. 2020 IEEE/CVF Conference on Computer Vision and Pattern Recognition (CVPR):4084-4094

7 Zhang J, Cui Z, Shi Z et al (2023) A robust and efficient AI assistant for breast tumor segmentation from DCE-MRI via a spatial-temporal framework. Patterns (N Y) 4:100826

8 Shi Z, Huang X, Cheng Z et al (2023) MRI-based Quantification of Intratumoral Heterogeneity for Predicting Treatment Response to Neoadjuvant Chemotherapy in Breast Cancer. Radiology 308:e222830

9 van Griethuysen JJM, Fedorov A, Parmar C et al (2017) Computational Radiomics System to Decode the Radiographic Phenotype. Cancer res 77:e104-e107

10 Natrajan R, Sailem H, Mardakheh FK et al (2016) Microenvironmental Heterogeneity Parallels Breast Cancer Progression: A Histology-Genomic Integration Analysis. PLoS Med 13:e1001961

11 Liu Y, Li Z, Xiong H, Gao X, Wu J, Wu S (2013) Understanding and enhancement of internal clustering validation measures. Ieee t cybernetics 43:982-994

**Table S1. MRI Acquisition Parameters from hospital in different cohorts.**

| **Dataset** | **Scanner** | **Sequence** | **TR/TE (ms)** | **FOV (mm)** | **Matrix** | **Section Thickness (mm)** | **Section Gap (mm)** | **No. of Sections** | **Flip Angle** | **No. of phase** |
| --- | --- | --- | --- | --- | --- | --- | --- | --- | --- | --- |
| **GZ dataset** | United Imaging 1.5T (uMR 560) | T1+C | 4.8/2.1 | 320×320 | 400×400 | 1.2 | 1.2 | 120 | 10° | 6 |
| **LZ dataset** | Philips 3.0T (Achieva) | T1+C | 3.2/1.6 | 360×360 | 256×256 | 1.5 | 1.5 | 150 | 10° | 10 |
| **DG dataset** | Philips 3.0T (Ingenia) | T1+C | 4.8/1.8 | 400×400 | 512×512 | 1.5 | 1.5 | 112 | 10° | 7 |
| **YT dataset** | GE 3.0T (Discovery MR750W) | T1+C | 6.6/1.7 | 360×360 | 512×512 | 2 | 0.7 | 104 | 12° | 8 |
| **YN dataset** | Philips 3.0T (Ingenia) | T1+C | 4.5/2.3 | 340×340 | 512×512 | 2 | 1.5 | 107 | 12° | 7 |

Note.—T1+C = contrast-enhanced T1-weighted imaging, FOV = field of view, TR = repetition time, TE = echo time, No. = number, ms = millisecond, mm = millimeter

**Table S2. Quantitative Evaluation of Optimal Cluster Number Based on BIC and CH Scores.**

| **Number of clusters** | **BIC Score** | **CH Score** |
| --- | --- | --- |
| 3 | 18278.01 | 574.51 |
| 4 | 19918.66 | 241.14 |
| 5 | 20142.82 | 250.99 |
| 6 | 19775.06 | 288.74 |

Abbreviations: BIC: Bayesian Information Criterion; CH: Calinski-Harabasz

**Table S****3.** **Clinicopathological characteristics of the patients from public datasets.**

| **Characteristics** | **ACRIN 6657 / I-SPY 1 cohort**  **(N=126)** | **ACRIN 6698 / I-SPY 2 cohort**  **(N=222)** |
| --- | --- | --- |
| **Age** | 48.2 (8.9) | 48.1 (10.6) |
| **LD at baseline (mm)** | 67.3 (29.1) | 42.2 (22.4) |
| **ER status** |  |  |
| Positive | 76 (60.3%) | NA |
| Negative | 50 (39.7%) | NA |
| **PR status** |  |  |
| Positive | 62 (49.2%) | NA |
| Negative | 64 (50.8%) | NA |
| **HER2 status** |  |  |
| Positive | 38 (30.2%) | NA |
| Negative | 88 (69.8%) | NA |
| **Molecular subtype** |  |  |
| HR+/HER2- | 59 (46.8%) | 97 (43.7%) |
| HER2+ | 38 (30.2%) | 57 (25.7%) |
| HR-/HER2- (TN) | 29 (23.0%) | 68 (30.6%) |
| **Response to treatment** |  |  |
| pCR | 30 (23.8%) | 54 (24.3%) |
| non-pCR | 96 (76.2%) | 168 (75.7%) |

Data are presented as number of patients, except for age and LD at baseline (mean [SD]). For categorical variables such as ER, PR, HER2, and molecular subtype, the first value is the count and the value in parentheses represents the percentage of total patients.

Note: The ER, PR, and HER2 status were not provided for the ACRIN 6698/I-SPY 2 cohort in the public dataset and are therefore marked as "NA".

Abbreviations: ER: estrogen receptor; PR: progesterone receptor; HER2: human epidermal growth factor receptor; HR: hormone receptor; NA= not applicable; SD: standard deviation.

**Table S4.** **Performances of deep learning models combined with different time and space for predicting pCR to NAC in different cohorts.**

| **Model** | **Cohorts** | **Accuracy** | **Sensitivity** | **Specificity** | **PPV** | **NPV** |
| --- | --- | --- | --- | --- | --- | --- |
| **T0 model** | **PC** | 0.625 (202/323) | 0.476 (40/84) | 0.678 (162/239) | 0.342 (40/117) | 0.786 (162/206) |
|  | **EVC total** | 0.518 (160/309) | 0.485 (48/99) | 0.533 (112/210) | 0.329 (48/146) | 0.687 (112/163) |
|  | EVC 1 | 0.537 (51/95) | 0.486 (17/35) | 0.567 (34/60) | 0.395 (17/43) | 0.654 (34/52) |
|  | EVC 2 | 0.534 (70/131) | 0.368 (14/38) | 0.602 (56/93) | 0.275 (14/51) | 0.700 (56/80) |
|  | EVC 3 | 0.470 (39/83) | 0.654 (17/26) | 0.386 (22/57) | 0.327 (17/52) | 0.710 (22/31) |
| **T1 model** | **PC** | 0.783 (253/323) | 0.440 (37/84) | 0.904 (216/239) | 0.617 (37/60) | 0.821 (216/263) |
|  | **EVC total** | 0.738 (228/309) | 0.465 (46/99) | 0.867 (182/210) | 0.622 (46/74) | 0.774 (182/235) |
|  | EVC 1 | 0.758 (72/95) | 0.629 (22/35) | 0.833 (50/60) | 0.688 (22/32) | 0.794 (50/63) |
|  | EVC 2 | 0.725 (95/131) | 0.237 (9/38) | 0.925 (86/93) | 0.562 (9/16) | 0.748 (86/115) |
|  | EVC 3 | 0.735 (61/83) | 0.577 (15/26) | 0.807 (46/57) | 0.577 (15/26) | 0.807 (46/57) |
| **T0 + T1 model** | **PC** | 0.901 (291/323) | 0.798 (67/84) | 0.937 (224/239) | 0.817 (67/82) | 0.929 (224/241) |
|  | **EVC total** | 0.767 (237/309) | 0.596 (59/99) | 0.848 (178/210) | 0.648 (59/91) | 0.817 (178/218) |
|  | EVC 1 | 0.758 (72/95) | 0.743 (26/35) | 0.767 (46/60) | 0.650 (26/40) | 0.836 (46/55) |
|  | EVC 2 | 0.771 (101/131) | 0.421 (16/38) | 0.914 (85/93) | 0.667 (16/24) | 0.794 (85/107) |
|  | EVC 3 | 0.771 (64/83) | 0.654 (17/26) | 0.825 (47/57) | 0.630 (17/27) | 0.839 (47/56) |
| **T0+Spatial model** | **PC** | 0.666 (215/323) | 0.512 (43/84) | 0.720 (172/239) | 0.391 (43/110) | 0.808 (172/213) |
|  | **EVC total** | 0.528 (163/309) | 0.465 (46/99) | 0.557 (117/210) | 0.331 (46/139) | 0.688 (117/170) |
|  | EVC 1 | 0.558 (53/95) | 0.486 (17/35) | 0.600 (36/60) | 0.415 (17/41) | 0.667 (36/54) |
|  | EVC 2 | 0.527 (69/131) | 0.368 (14/38) | 0.591 (55/93) | 0.269 (14/52) | 0.696 (55/79) |
|  | EVC 3 | 0.494 (41/83) | 0.577 (15/26) | 0.456 (26/57) | 0.326 (15/46) | 0.703 (26/37) |
| **T1+Spatial model** | **PC** | 0.848 (274/323) | 0.798 (67/84) | 0.866 (207/239) | 0.677 (67/99) | 0.924 (207/224) |
|  | **EVC total** | 0.748 (231/309) | 0.566 (56/99) | 0.833 (175/210) | 0.615 (56/91) | 0.803 (175/218) |
|  | EVC 1 | 0.779 (74/95) | 0.629 (22/35) | 0.867 (52/60) | 0.733 (22/30) | 0.800 (52/65) |
|  | EVC 2 | 0.733 (96/131) | 0.447 (17/38) | 0.850 (79/93) | 0.548 (17/31) | 0.790 (79/100) |
|  | EVC 3 | 0.735 (61/83) | 0.654 (17/26) | 0.772 (44/57) | 0.567 (17/30) | 0.830 (44/53) |
| **STI model** | **PC** | 0.907 (293/323) | 0.952 (80/84) | 0.891 (213/239) | 0.755 (80/106) | 0.982 (213/217) |
|  | **EVC total** | 0.806 (249/309) | 0.859 (85/99) | 0.781 (164/210) | 0.649 (85/131) | 0.921 (164/178) |
|  | EVC 1 | 0.821 (78/95) | 0.943 (33/35) | 0.750 (45/60) | 0.688 (33/48) | 0.957 (45/47) |
|  | EVC 2 | 0.809 (106/131) | 0.763 (29/38) | 0.828 (77/93) | 0.644 (29/45) | 0.895 (77/86) |
|  | EVC 3 | 0.783 (65/83) | 0.885 (23/26) | 0.737 (42/57) | 0.605 (23/38) | 0.933 (42/45) |

Note: Values are presented as percentages, with numerators and denominators shown in parentheses. For example, accuracy is shown as correct predictions / total cases; sensitivity as true positives / actual positives; specificity as true negatives / actual negatives; PPV as true positives / predicted positives; and NPV as true negatives / predicted negatives.

Abbreviations: pCR, pathological complete response; PPV: positive predictive value; NPV: negative predictive value; PC, primary cohort; EVC, external validation cohort; STI model: Spatiotemporal interaction model.

**Table S5. Performances of longitudinal time (T0+T1) only spatial model for predicting pCR to NAC in different cohorts.**

| **Model** | **Cohorts** | **AUC (95% CI)** | **AP (95% CI)** | **Accuracy (95% CI)** | **Sensitivity (95% CI)** | **Specificity (95% CI)** | **PPV (95% CI)** | **NPV (95% CI)** |
| --- | --- | --- | --- | --- | --- | --- | --- | --- |
| **T0 + T1 only spatial model** | **PC** | 0.882 (0.833~0.931) | 0.764 (0.670~0.846) | 0.820 (0.774~0.861) | 0.798 (0.696~0.877) | 0.828 (0.775~0.874) | 0.620 (0.522~0.712) | 0.921 (0.876~0.953) |
|  | **EVC total** | 0.810 (0.754~0.866) | 0.631 (0.536~0.737) | 0.741 (0.688~0.789) | 0.697 (0.596~0.785) | 0.762 (0.698~0.818) | 0.580 (0.486~0.670) | 0.842 (0.782~0.891) |
|  | EVC 1 | 0.818 (0.723~0.912) | 0.738 (0.584~0.874) | 0.716 (0.614~0.804) | 0.743 (0.567~0.875) | 0.700 (0.568~0.812) | 0.591 (0.432~0.737) | 0.824 (0.691~0.916) |
|  | EVC 2 | 0.791 (0.698~0.884) | 0.568 (0.421~0.740) | 0.748 (0.665~0.820) | 0.605 (0.434~0.760) | 0.806 (0.711~0.881) | 0.561 (0.397~0.715) | 0.833 (0.740~0.904) |
|  | EVC 3 | 0.827 (0.721~0.932) | 0.644 (0.457~0.826) | 0.759 (0.653~0.846) | 0.769 (0.564~0.910) | 0.754 (0.622~0.859) | 0.588 (0.407~0.754) | 0.878 (0.752~0.954) |

Abbreviations: pCR, pathological complete response; AUC: area under the receiver operating characteristics curve; AP: area under the P-R(precision-recall) cruve; PPV: positive predictive value; NPV: negative predictive value; PC, primary cohort; EVC, external validation cohort; 95% CI, 95% confidence interval; STI model: Spatiotemporal interaction model.

**Table S6. Performances of STI models for predicting pCR to NAC in various molecular subtypes and clinical T stage (with raw numerator/denominator values).**

| **Molecular subtype** | **Cohorts** | **Accuracy** | **Sensitivity** | **Specificity** | **PPV** | **NPV** |
| --- | --- | --- | --- | --- | --- | --- |
| **HR+/HER2-** | **PC** | 0.954 (145/152) | 1.000 (8/8) | 0.951 (137/144) | 0.533 (8/15) | 1.000 (137/137) |
|  | **EVC total** | 0.858 (97/113) | 0.818 (9/11) | 0.863 (88/102) | 0.391 (9/23) | 0.978 (88/90) |
|  | EVC 1 | 0.853 (29/34) | 1.000 (3/3) | 0.839 (26/31) | 0.375 (3/8) | 1.000 (26/26) |
|  | EVC 2 | 0.911 (41/45) | 1.000 (1/1) | 0.909 (40/44) | 0.200 (1/5) | 1.000 (40/40) |
|  | EVC 3 | 0.794 (27/34) | 0.714 (5/7) | 0.815 (22/27) | 0.500 (5/10) | 0.917 (22/24) |
| **HER2+** | **PC** | 0.905 (124/137) | 0.942 (65/69) | 0.868 (59/68) | 0.878 (65/74) | 0.937 (59/63) |
|  | **EVC total** | 0.766 (121/158) | 0.910 (71/78) | 0.625 (50/80) | 0.703 (71/101) | 0.877 (50/57) |
|  | EVC 1 | 0.776 (38/49) | 0.931 (27/29) | 0.550 (11/20) | 0.750 (27/36) | 0.846 (11/13) |
|  | EVC 2 | 0.787 (59/75) | 0.875 (28/32) | 0.721 (31/43) | 0.700 (28/40) | 0.886 (31/35) |
|  | EVC 3 | 0.706 (24/34) | 0.941 (16/17) | 0.471 (8/17) | 0.640 (16/25) | 0.889 (8/9) |
| **HR-/HER2- (TNBC)** | **PC** | 0.912 (31/34) | 1.000 (7/7) | 0.889 (24/27) | 0.700 (7/10) | 1.000 (24/24) |
|  | **EVC total** | 0.868 (33/38) | 0.800 (8/10) | 0.893 (25/28) | 0.727 (8/11) | 0.926 (25/27) |
|  | EVC 1 | 0.833 (10/12) | 1.000 (3/3) | 0.778 (7/9) | 0.600 (3/5) | 1.000 (7/7) |
|  | EVC 2 | 0.818 (9/11) | 0.600 (3/5) | 1.000 (6/6) | 1.000 (3/3) | 0.750 (6/8) |
|  | EVC 3 | 0.933 (14/15) | 1.000 (2/2) | 0.923 (12/13) | 0.667 (2/3) | 1.000 (12/12) |
| **Clinical T stage** |  |  |  |  |  |  |
| **T1-2** | **PC** | 0.914 (201/220) | 0.902 (55/61) | 0.918 (146/159) | 0.809 (55/68) | 0.961 (146/152) |
|  | **EVC total** | 0.778 (165/212) | 0.850 (68/80) | 0.735 (97/132) | 0.660 (68/103) | 0.890 (97/109) |
|  | EVC 1 | 0.813 (61/75) | 0.935 (29/31) | 0.727 (32/44) | 0.707 (29/41) | 0.941 (32/34) |
|  | EVC 2 | 0.773 (68/88) | 0.733 (22/30) | 0.793 (46/58) | 0.647 (22/34) | 0.852 (46/54) |
|  | EVC 3 | 0.735 (36/49) | 0.895 (17/19) | 0.633 (19/30) | 0.607 (17/28) | 0.905 (19/21) |
| **T3-4** | **PC** | 0.932 (96/103) | 1.000 (23/23) | 0.912 (73/80) | 0.767 (23/30) | 1.000 (73/73) |
|  | **EVC total** | 0.866 (84/97) | 0.895 (17/19) | 0.859 (67/78) | 0.607 (17/28) | 0.971 (67/69) |
|  | EVC 1 | 0.850 (17/20) | 1.000 (4/4) | 0.812 (13/16) | 0.571 (4/7) | 1.000 (13/13) |
|  | EVC 2 | 0.884 (38/43) | 0.875 (7/8) | 0.886 (31/35) | 0.636 (7/11) | 0.969 (31/32) |
|  | EVC 3 | 0.853 (29/34) | 0.857 (6/7) | 0.852 (23/27) | 0.600 (6/10) | 0.958 (23/24) |

Note: Values are presented as percentages, with numerators and denominators shown in parentheses. For example, accuracy is shown as correct predictions / total cases; sensitivity as true positives / actual positives; specificity as true negatives / actual negatives; PPV as true positives / predicted positives; and NPV as true negatives / predicted negatives.

Abbreviations: pCR: pathological complete response; HR: hormone receptor; HER2: human epidermal growth factor receptor; PC, primary cohort; EVC, external validation cohort; STI model: Spatiotemporal interaction model.

**Table S7. The ablation studies of no spatial or time attention for STI model in different cohorts (with raw numerator/denominator values).**

| **Model** | **Cohorts** | **Accuracy** | **Sensitivity** | **Specificity** | **PPV** | **NPV** |
| --- | --- | --- | --- | --- | --- | --- |
| **No Spatial Attention** | **PC** | 0.777 (251/323) | 0.679 (57/84) | 0.812 (194/239) | 0.559 (57/102) | 0.878 (194/221) |
|  | **EVC total** | 0.706 (218/309) | 0.768 (76/99) | 0.676 (142/210) | 0.528 (76/144) | 0.861 (142/165) |
|  | EVC 1 | 0.632 (60/95) | 0.829 (29/35) | 0.517 (31/60) | 0.500 (29/58) | 0.838 (31/37) |
|  | EVC 2 | 0.740 (97/131) | 0.632 (24/38) | 0.785 (73/93) | 0.546 (24/44) | 0.839 (73/87) |
|  | EVC 3 | 0.735 (61/83) | 0.885 (23/26) | 0.667 (38/57) | 0.548 (23/42) | 0.927 (38/41) |
| **No Time Attention** | **PC** | 0.845 (273/323) | 0.643 (54/84) | 0.916 (219/239) | 0.730 (54/74) | 0.880 (219/249) |
|  | **EVC total** | 0.751 (232/309) | 0.515 (51/99) | 0.862 (181/210) | 0.638 (51/80) | 0.790 (181/229) |
|  | EVC 1 | 0.737 (70/95) | 0.571 (20/35) | 0.833 (50/60) | 0.667 (20/30) | 0.769 (50/65) |
|  | EVC 2 | 0.702 (92/131) | 0.342 (13/38) | 0.849 (79/93) | 0.481 (13/27) | 0.760 (79/104) |
|  | EVC 3 | 0.843 (70/83) | 0.692 (18/26) | 0.912 (52/57) | 0.783 (18/23) | 0.867 (52/60) |

Note: Values are presented as percentages, with numerators and denominators shown in parentheses. For example, accuracy is shown as correct predictions / total cases; sensitivity as true positives / actual positives; specificity as true negatives / actual negatives; PPV as true positives / predicted positives; and NPV as true negatives / predicted negatives.

Abbreviations: AUC: area under the receiver operating characteristics curve; PC, primary cohort; EVC, external validation cohort; STI model: Spatiotemporal interaction model.

**Table S8.** **Performance comparison of** **the STI model without preprocessing or** **pretraining across multiple cohorts (with 95% confidence intervals).**

| **Model** | **Cohorts** | **AUC (95% CI)** | **Accuracy (95% CI)** | **Sensitivity (95% CI)** | **Specificity (95% CI)** | **PPV (95% CI)** | **NPV (95% CI)** | **p value*** |
| --- | --- | --- | --- | --- | --- | --- | --- | --- |
| **No**  **Preprocessing** | PC | 0.918 (0.876~0.960) | 0.861 (0.818~0.897) | 0.869 (0.778~0.933) | 0.858 (0.807~0.899) | 0.682 (0.585~0.769) | 0.949 (0.911~0.974) | 0.0007 |
|  | EVC total | 0.803 (0.747~0.860) | 0.712 (0.658~0.762) | 0.747 (0.650~0.829) | 0.695 (0.628~0.757) | 0.536 (0.449~0.621) | 0.854 (0.792~0.903) | <0.0001 |
|  | EVC 1 | 0.798 (0.699~0.896) | 0.684 (0.581~0.776) | 0.829 (0.664~0.934) | 0.600 (0.465~0.724) | 0.547 (0.404~0.684) | 0.857 (0.715~0.946) | 0.0014 |
|  | EVC 2 | 0.797 (0.705~0.889) | 0.710 (0.624~0.786) | 0.658 (0.486~0.804) | 0.731 (0.629~0.818) | 0.500 (0.355~0.645) | 0.840 (0.741~0.912) | 0.0041 |
|  | EVC 3 | 0.802 (0.691~0.913) | 0.747 (0.640~0.836) | 0.769 (0.564~0.910) | 0.737 (0.603~0.845) | 0.571 (0.394~0.737) | 0.875 (0.748~0.953) | 0.013 |
| **No**  **Pretraining** | PC | 0.916 (0.874~0.958) | 0.870 (0.828~0.905) | 0.774 (0.670~0.858) | 0.904 (0.859~0.938) | 0.739 (0.634~0.827) | 0.919 (0.877~0.951) | 0.0019 |
|  | EVC total | 0.797 (0.740~0.854) | 0.751 (0.699~0.798) | 0.667 (0.565~0.758) | 0.790 (0.729~0.843) | 0.600 (0.502~0.692) | 0.834 (0.775~0.883) | 0.0001 |
|  | EVC 1 | 0.798 (0.699~0.896) | 0.737 (0.636~0.822) | 0.686 (0.507~0.831) | 0.767 (0.640~0.866) | 0.632 (0.460~0.782) | 0.807 (0.681~0.900) | 0.0082 |
|  | EVC 2 | 0.797 (0.705~0.889) | 0.763 (0.681~0.833) | 0.579 (0.408~0.737) | 0.839 (0.748~0.907) | 0.595 (0.421~0.752) | 0.830 (0.738~0.899) | 0.024 |
|  | EVC 3 | 0.795 (0.682~0.908) | 0.747 (0.640~0.836) | 0.769 (0.564~0.910) | 0.737 (0.603~0.845) | 0.571 (0.394~0.737) | 0.875 (0.748~0.953) | 0.0079 |

Abbreviations: AUC: area under the receiver operating characteristics curve; PC, primary cohort; EVC, external validation cohort; 95% CI, 95% confidence interval; STI model: Spatiotemporal interaction model.

* The p-value represents the comparison between the AUC of the STI model without preprocessing or pretraining and the AUC of the STI model.

**Table S9. Performance comparison of the STI model without preprocessing or pretraining across multiple cohorts (with raw numerator/denominator values).**

| **Model** | **Cohorts** | **Accuracy** | **Sensitivity** | **Specificity** | **PPV** | **NPV** |
| --- | --- | --- | --- | --- | --- | --- |
| **No Preprocessing** | **PC** | 0.861 (278/323) | 0.869 (73/84) | 0.858 (205/239) | 0.682 (73/107) | 0.949 (205/216) |
|  | **EVC total** | 0.712 (220/309) | 0.747 (74/99) | 0.695 (146/210) | 0.536 (74/138) | 0.854 (146/171) |
|  | EVC 1 | 0.684 (65/95) | 0.829 (29/35) | 0.600 (36/60) | 0.547 (29/53) | 0.857 (36/42) |
|  | EVC 2 | 0.710 (93/131) | 0.658 (25/38) | 0.731 (68/93) | 0.500 (25/50) | 0.840 (68/81) |
|  | EVC 3 | 0.747 (62/83) | 0.769 (20/26) | 0.737 (42/57) | 0.571 (20/35) | 0.875 (42/48) |
| **No Pretraining** | **PC** | 0.870 (281/323) | 0.774 (65/84) | 0.904 (216/239) | 0.739 (65/88) | 0.919 (216/235) |
|  | **EVC total** | 0.751 (232/309) | 0.667 (66/99) | 0.790 (166/210) | 0.600 (66/110) | 0.834 (166/199) |
|  | EVC 1 | 0.737 (70/95) | 0.686 (24/35) | 0.767 (46/60) | 0.632 (24/38) | 0.807 (46/57) |
|  | EVC 2 | 0.763 (100/131) | 0.579 (22/38) | 0.839 (78/93) | 0.595 (22/37) | 0.830 (78/94) |
|  | EVC 3 | 0.747 (62/83) | 0.769 (20/26) | 0.737 (42/57) | 0.571 (20/35) | 0.875 (42/48) |

Note: Values are presented as percentages, with numerators and denominators shown in parentheses. For example, accuracy is shown as correct predictions / total cases; sensitivity as true positives / actual positives; specificity as true negatives / actual negatives; PPV as true positives / predicted positives; and NPV as true negatives / predicted negatives.

Abbreviations: AUC: area under the receiver operating characteristics curve; PC, primary cohort; EVC, external validation cohort; STI model: Spatiotemporal interaction model.

**Table S10.** **The performance of clinical model and STI model combining with clinical data (with raw numerator/denominator values).**

| **Model** | **Cohorts** | **Accuracy** | **Sensitivity** | **Specificity** | **PPV** | **NPV** |
| --- | --- | --- | --- | --- | --- | --- |
| **Clinical model** | **PC** | 0.810 (260/321) | 0.659 (54/82) | 0.862 (206/239) | 0.621 (54/87) | 0.880 (206/234) |
|  | **EVC total** | 0.770 (241/313) | 0.604 (61/101) | 0.849 (180/212) | 0.656 (61/93) | 0.818 (180/220) |
|  | EVC 1 | 0.758 (72/95) | 0.514 (18/35) | 0.900 (54/60) | 0.750 (18/24) | 0.761 (54/71) |
|  | EVC 2 | 0.770 (104/135) | 0.650 (26/40) | 0.821 (78/95) | 0.605 (26/43) | 0.848 (78/92) |
|  | EVC 3 | 0.783 (65/83) | 0.654 (17/26) | 0.842 (48/57) | 0.654 (17/26) | 0.842 (48/57) |
| **STI model + Clinical data** | **PC** | 0.931 (299/321) | 0.951 (78/82) | 0.925 (221/239) | 0.812 (78/96) | 0.982 (221/225) |
|  | **EVC total** | 0.812 (251/309) | 0.838 (83/99) | 0.800 (168/210) | 0.664 (83/125) | 0.913 (168/184) |
|  | EVC 1 | 0.800 (76/95) | 0.886 (31/35) | 0.750 (45/60) | 0.674 (31/46) | 0.918 (45/49) |
|  | EVC 2 | 0.832 (109/131) | 0.816 (31/38) | 0.839 (78/93) | 0.674 (31/46) | 0.918 (78/85) |
|  | EVC 3 | 0.807 (67/83) | 0.846 (22/26) | 0.789 (45/57) | 0.647 (22/34) | 0.918 (45/49) |

Note: Values are presented as percentages, with numerators and denominators shown in parentheses. For example, accuracy is shown as correct predictions / total cases; sensitivity as true positives / actual positives; specificity as true negatives / actual negatives; PPV as true positives / predicted positives; and NPV as true negatives / predicted negatives.

Abbreviations: AUC: area under the receiver operating characteristics curve; PC, primary cohort; EVC, external validation cohort; 95% CI, 95% confidence interval; STI model: Spatiotemporal interaction model.

**Table S11. Performances of STI model for predicting pCR to NAC in public datasets.**

| **Model** | **Cohorts** | **AUC (95% CI)** | **Accuracy (95% CI)** | **Sensitivity (95% CI)** | **Specificity (95% CI)** |
| --- | --- | --- | --- | --- | --- |
| **STI model** | ACRIN 6657 / I-SPY 1 cohort | 0.762 (0.655-0.870) | 0.595 (0.504-0.682) | 0.833(0.653-0.944) | 0.521 (0.416-0.624) |
|  | ACRIN 6698 / I-SPY 2 cohort | 0.803 (0.728-0.878) | 0.635 (0.568-0.699) | 0.926 (0.821-0.979) | 0.542 (0.463-0.619) |

Note: 95% confidence intervals for AUC were calculated using the DeLong method. 95% confidence intervals for sensitivity, specificity and accuracy were calculated using the exact Clopper-Pearson method.

Abbreviations: pCR: pathological complete response; AUC: area under the receiver operating characteristics curve; 95% CI, 95% confidence interval; STI model: Spatiotemporal interaction model.


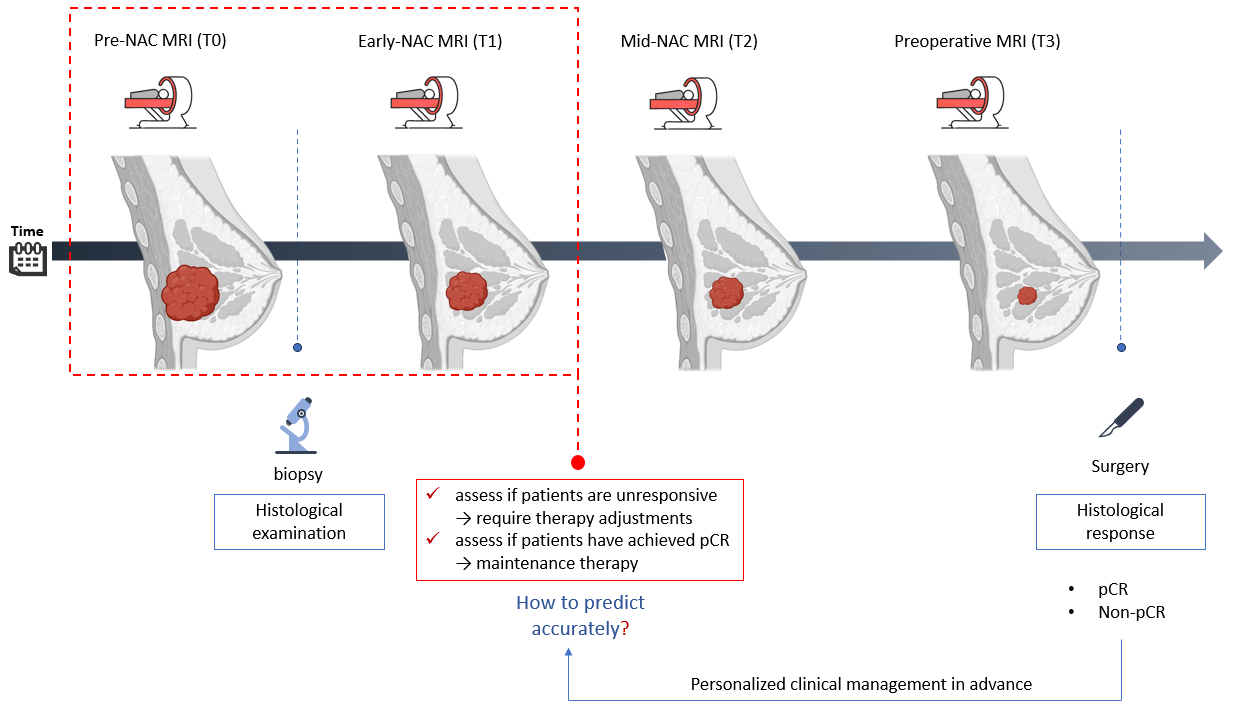


**Fig. S1:** **Longitudinal MRI Acquisition Timeline for NAC Response Assessment.** MRI scans are performed at multiple time points: Pre-NAC (T0), Early-NAC (T1), Mid-NAC (T2), and Preoperative MRI (T3), corresponding to different chemotherapy cycles. Early MRI-based response assessment (T0, T1) is crucial for identifying non-responders who may require treatment adjustments and patients achieving pCR who may benefit from maintenance therapy, enabling personalized clinical management in advance.

Note: NAC=neoadjuvant chemotherapy.


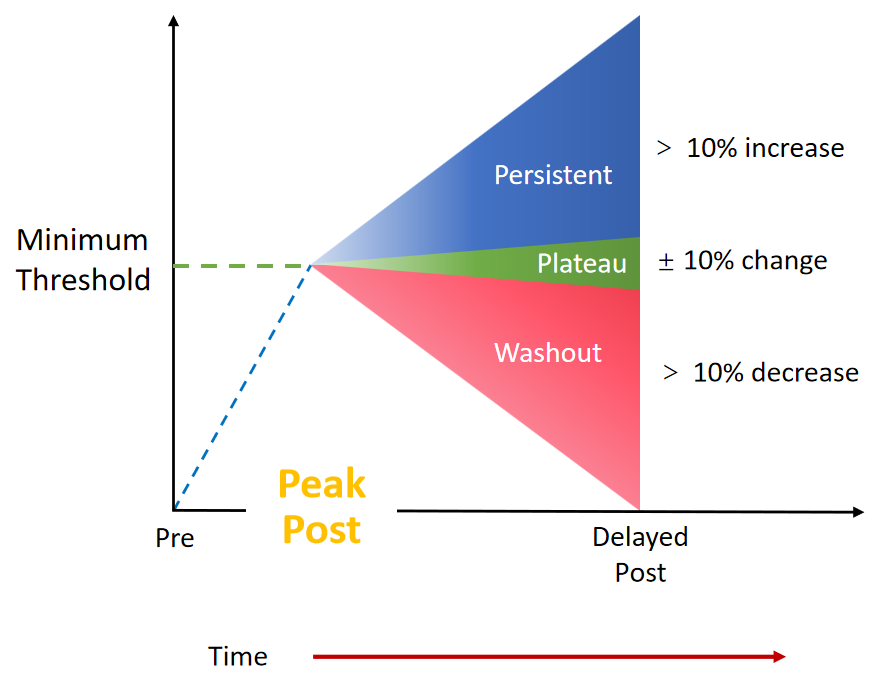


**Fig. S2: DCE-MRI kinetic enhancement patterns.** The diagram illustrates the three post-contrast enhancement curve types—Persistent, Plateau, and Washout—based on the percentage change in signal intensity following the peak enhancement phase ("Peak Post"). These classifications are consistent with the BI-RADS guidelines, which define the peak enhancement phase as occurring approximately 60–120 seconds after contrast injection.


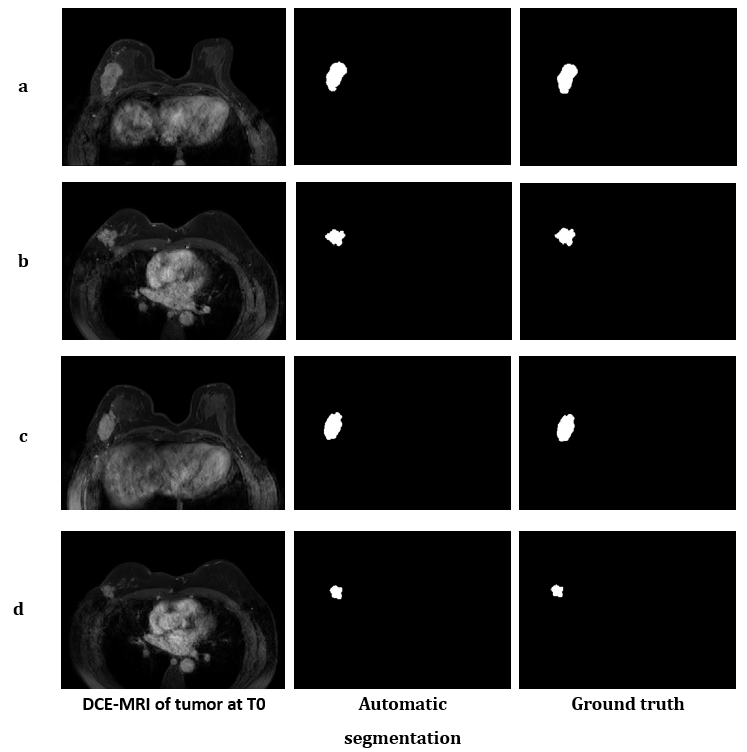


**Fig. S3: Segmentation results.** a and b show the pre- and post-treatment images (T0 and T1) with corresponding segmentation results for one patient, while c and d display the same for another patient undergoing NAC treatment.


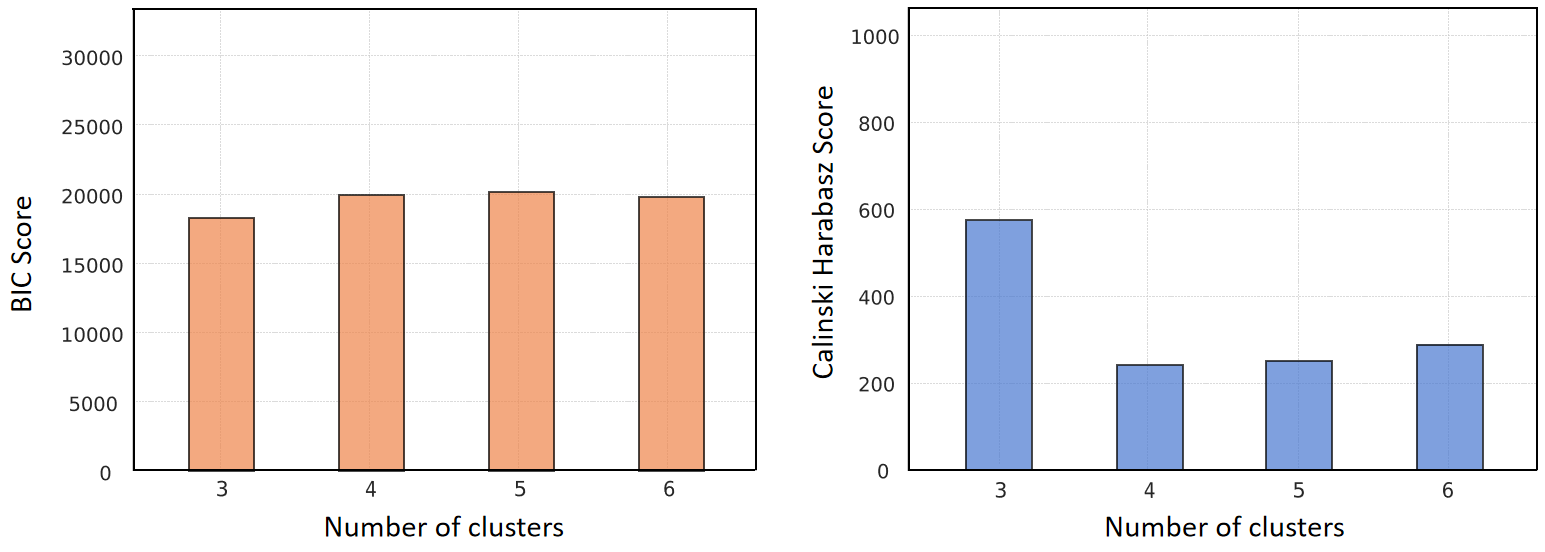


**Fig. S4: Determination of optimal cluster number using BIC and CH scores.** Bar plots display the Bayesian Information Criterion (BIC) scores (left) and Calinski-Harabasz (CH) scores (right) for different numbers of tumor subregions (clusters). A lower BIC score and a higher CH score indicate better clustering performance.


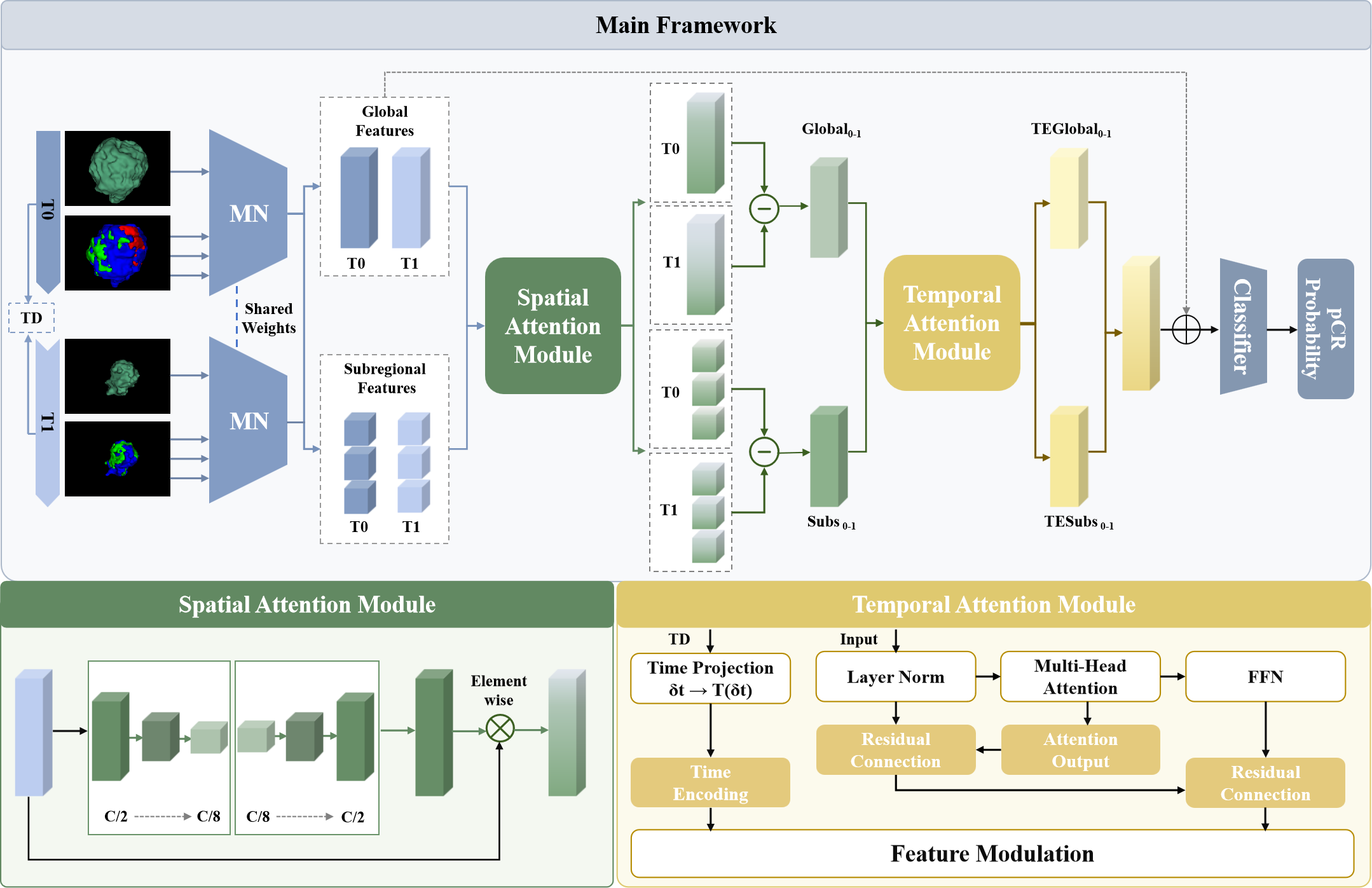


**Fig. S5: STI Model Architecture.** This figure illustrates the architecture of the STI model used for predicting pCR in breast cancer patients based on MRI data before and during NAC. **Main Framework:** The model processes MRI images from two time points (T0: Pre-NAC, T1: Early-NAC) by extracting global features and subregional features at each time point. **Spatial Attention Module:** This module captures spatial features by using a CNN to extract important tumor-related information from the images at both time points. Specifically, convolution layers are used to reduce channel dimension (C → C/8), apply ReLU activation, and restore dimension (C/8 → C), followed by a sigmoid function to generate attention maps. These attention maps are then applied element-wise multiplication to the input features for recalibration. **Temporal Attention Module:** This module captures the dynamic changes of the tumor over time (T0 to T1). The core idea is to integrate time interval information into feature difference representation through attention mechanism. Specifically, the module first extracts differential representations from the image features before and after treatment, which are used as queries (Q) and interacted with the time representations generated by the neural network encoded treatment time intervals as keys (K) and values (V). Through this design, the model can adaptively focus on relevant temporal information based on the characteristics of feature changes. These temporal differences are combined with the image feature differences, and the output is refined using residual connections for smooth integration of the features, enhancing the model’s ability to handle both spatial and temporal dynamics. **Classifier:** After the attention mechanisms, the final features are passed to a classifier to predict the likelihood of achieving pCR. The output of the classifier is the probability of pCR for each patient.

Note: TD = time difference; MN = Refers to the model backbone pre-trained using MedicalNet weights based on the ResNet18 model; pCR = pathological complete response.


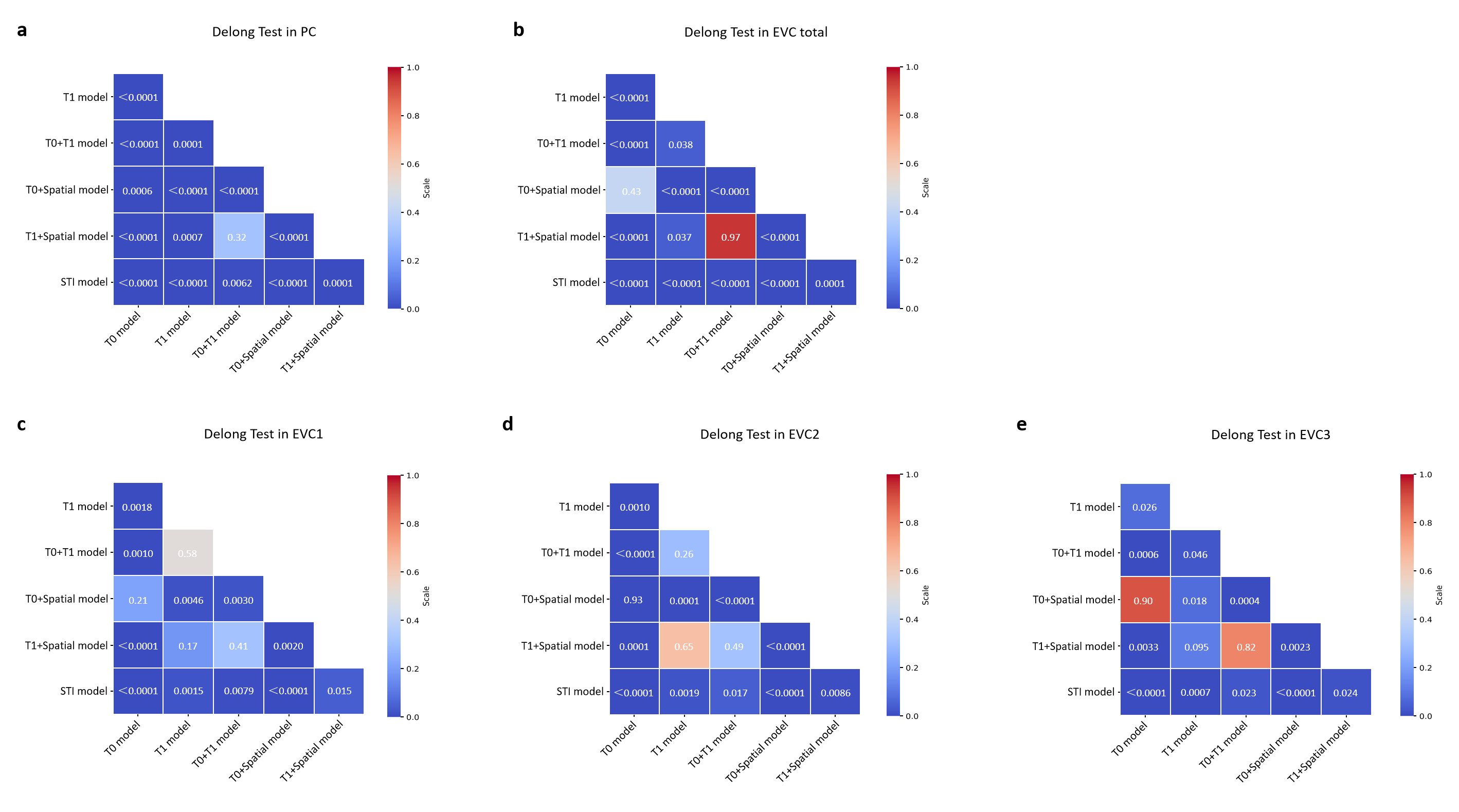


**Fig. S6: Comparison of AUC p-values between models.** Panels (a)-(e) show the pairwise Delong test results for AUC comparisons between different models (T0 model, T1 model, T0+Spatial model, T1+Spatial model, T0+T1 model, and STI model) across the primary and external validation cohorts (PC, EVC total, EVC1, EVC2, and EVC3). p-values < 0.05 indicate statistically significant differences in model performance.

Note: PC = primary cohort; EVC = external validated cohorts; STI = Spatiotemporal interaction model.


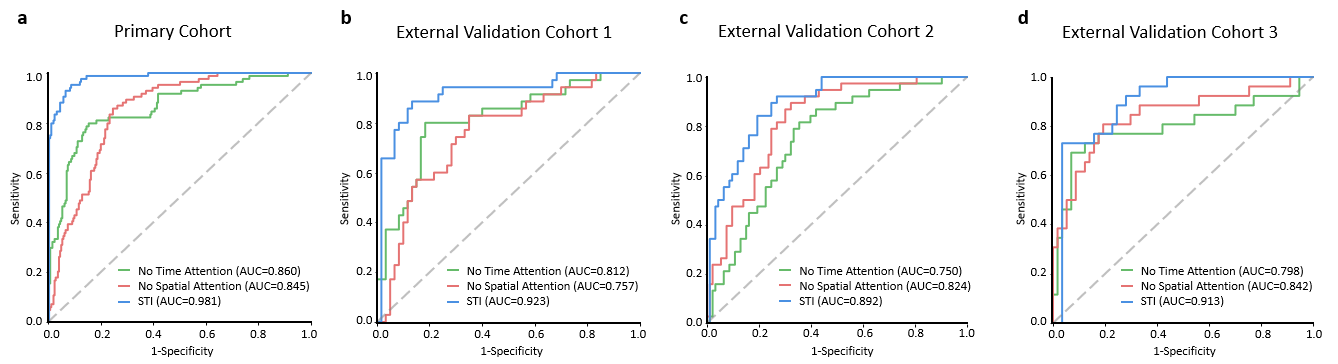


**Fig. S7:** **Ablation study of spatial and temporal attention in the STI model.** ROC curves compare the full STI model (blue) with model lacking spatial attention (red) or temporal attention (green) across the PC (a), EVC1 (b), EVC2 (c) and EVC3 (d).

Note: PC = primary cohort; EVC = external validated cohorts; STI = Spatiotemporal interaction model.


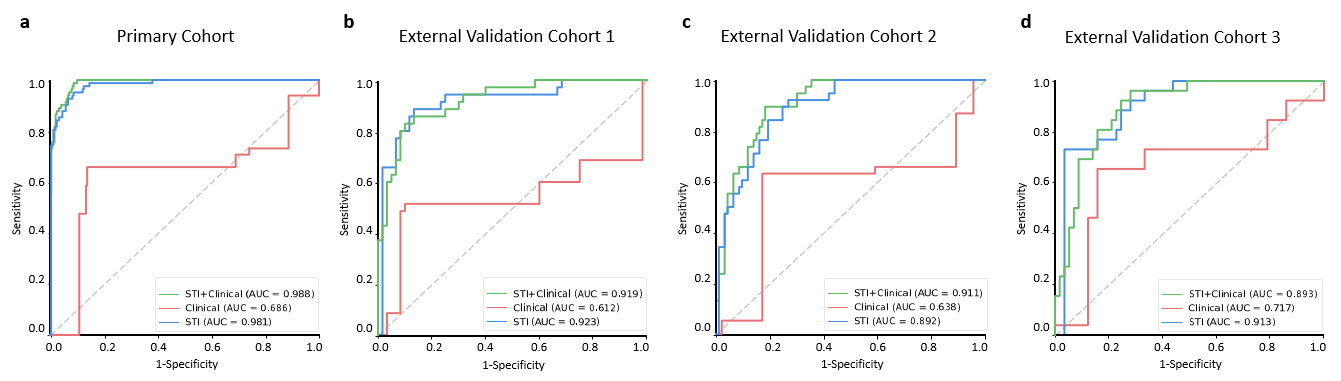


**Fig. S8: Performance Comparison of the STI Model with Clinical Models and STI+Clinical Model.** (a–d) Comparisons between the STI model, clinical model, and STI+clinical model across the PC (a), EVC1 (b), EVC2 (c) and EVC3 (d).

Note: PC = primary cohort; EVC = external validated cohorts; STI = Spatiotemporal interaction model.


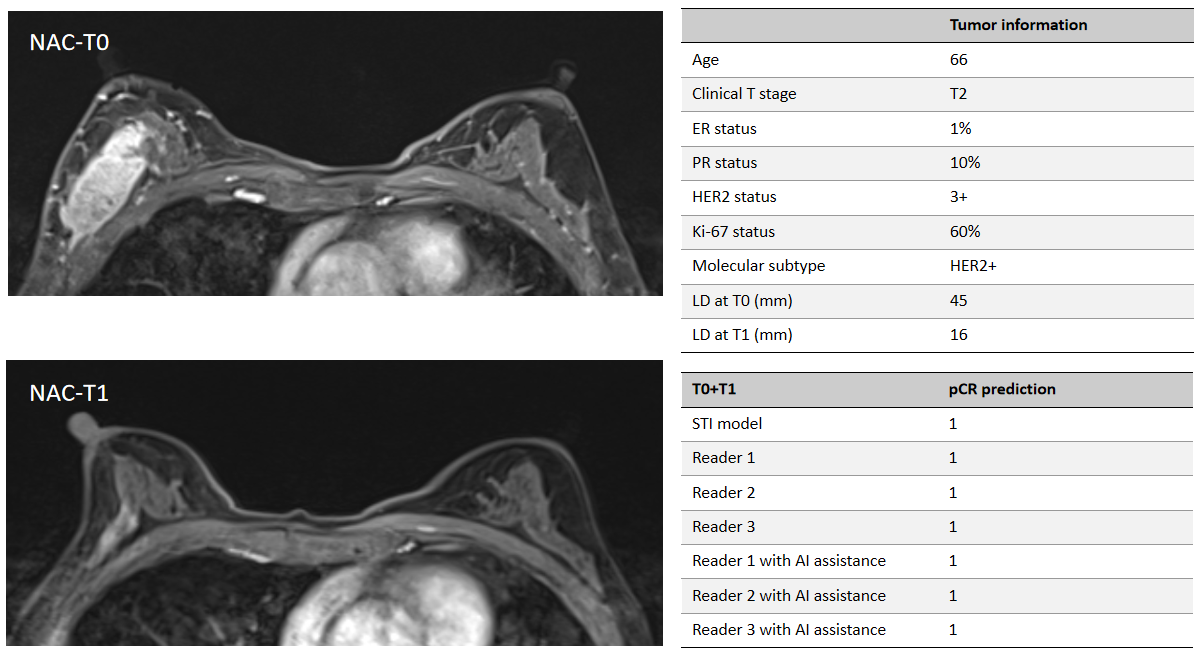


**Fig. S9: Case A.** This case illustrates a typical true-positive scenario where both the STI model and all three radiologists (with and without AI assistance) correctly predicted pCR. The tumor exhibited a marked reduction in size from 45 mm to 16 mm after NAC. The patient had a HER2-positive subtype, which is typically associated with good treatment response. This consistency demonstrates the strong alignment between model and expert assessments under classic pCR imaging patterns.


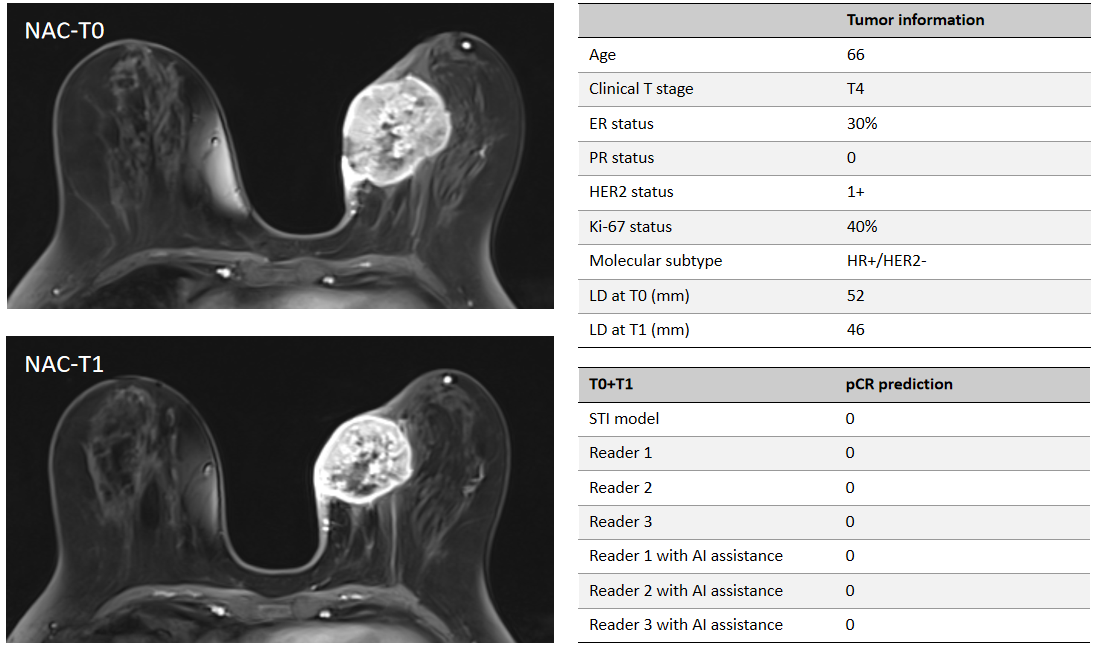


**Fig. S10: Case B.** An example of a consistent true-negative prediction across the STI model and radiologists. The tumor showed minimal change in size (52 mm to 46 mm) and enhancement pattern, with an HR+/HER2- subtype, often associated with lower chemosensitivity. All human readers and the model correctly identified this patient as non-pCR.


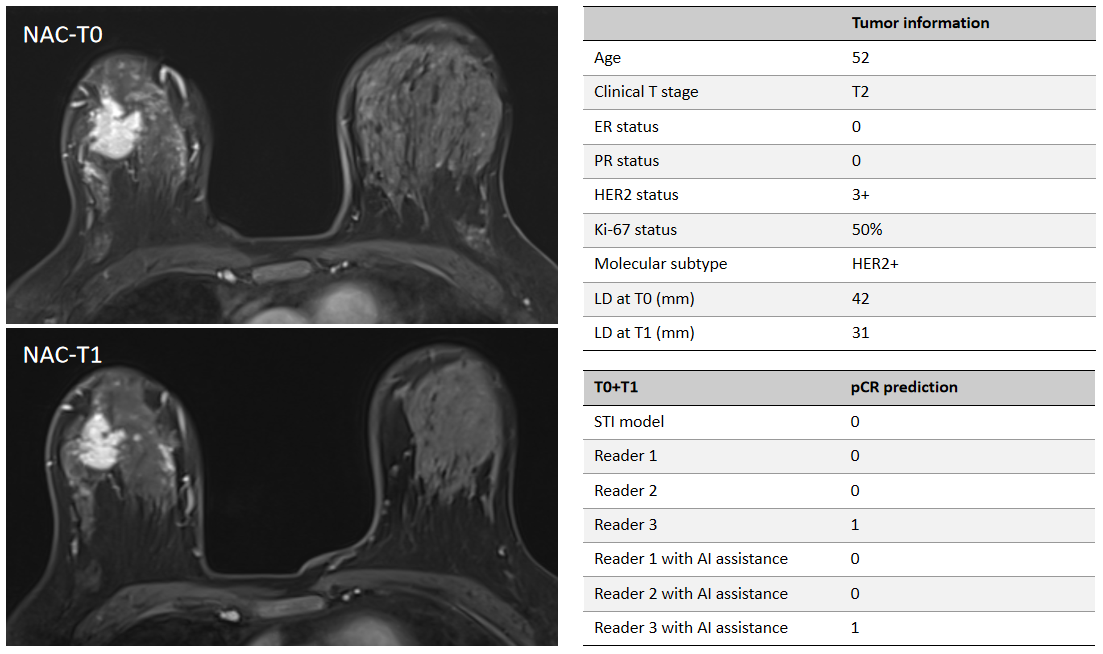


**Fig. S11: Case C.** This case represents a false-negative prediction: both the STI model and two radiologists (with and without AI assistance) misclassified a pCR case as non-pCR. The tumor showed slight shrinkage (42 mm to 31 mm). The ambiguity in the imaging features of this case led to misjudgments by both the radiologists and the model, highlighting the challenges in diagnosing subtle responders.


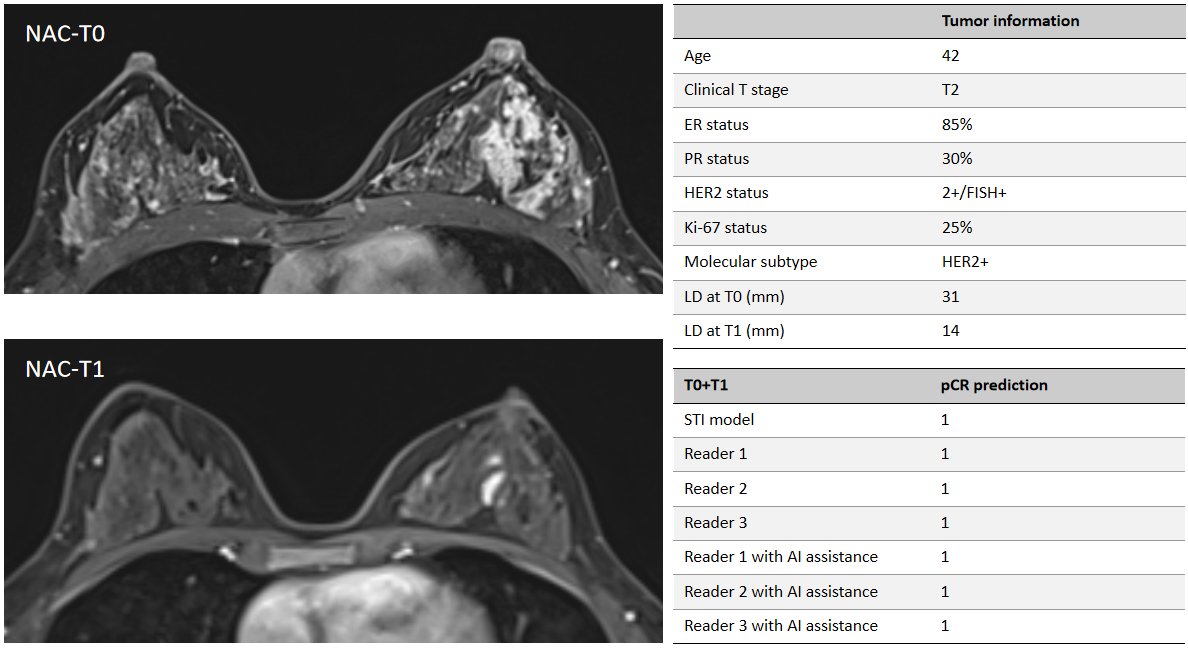


**Fig. S12: Case D.** A challenging case where the patient was non-pCR but misclassified as pCR by both the STI model and all radiologists. The imaging exhibited noticeable shrinkage (31 mm to 14 mm).


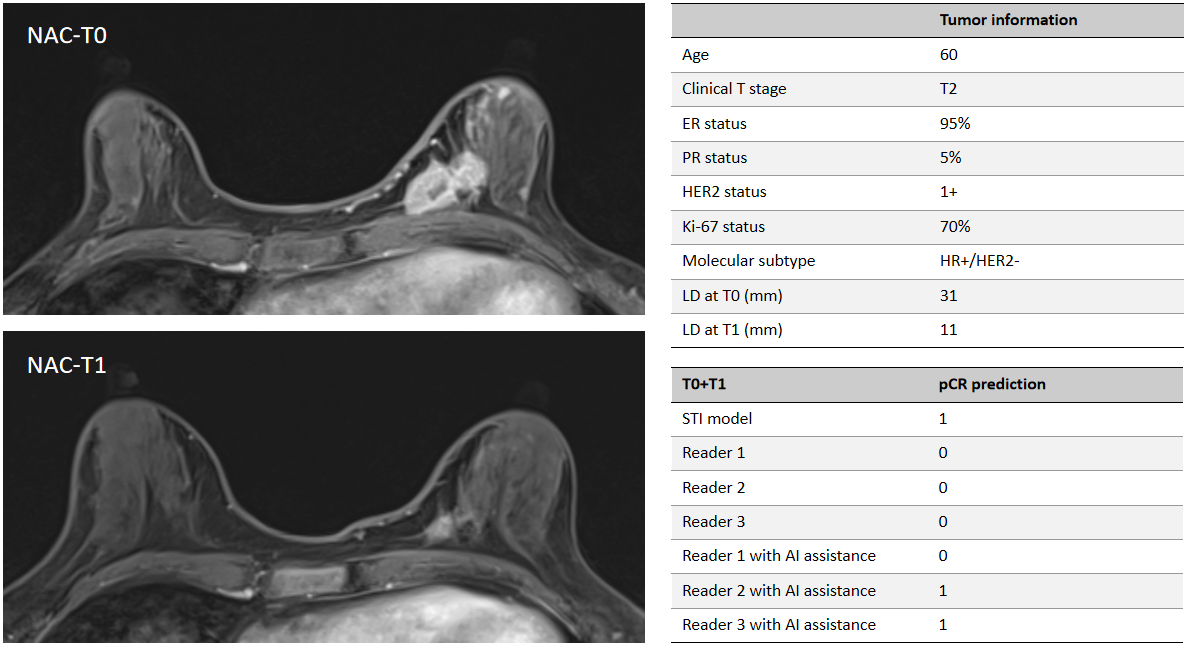


**Fig. S13: Case E.** This case demonstrates the benefit of AI assistance. Initially, all radiologists misclassified this patient as non-pCR based on the HR+/HER2- subtype. However, with the aid of the STI model, two out of three radiologists revised their decisions and correctly identified the patient as pCR, showcasing human-AI collaboration.


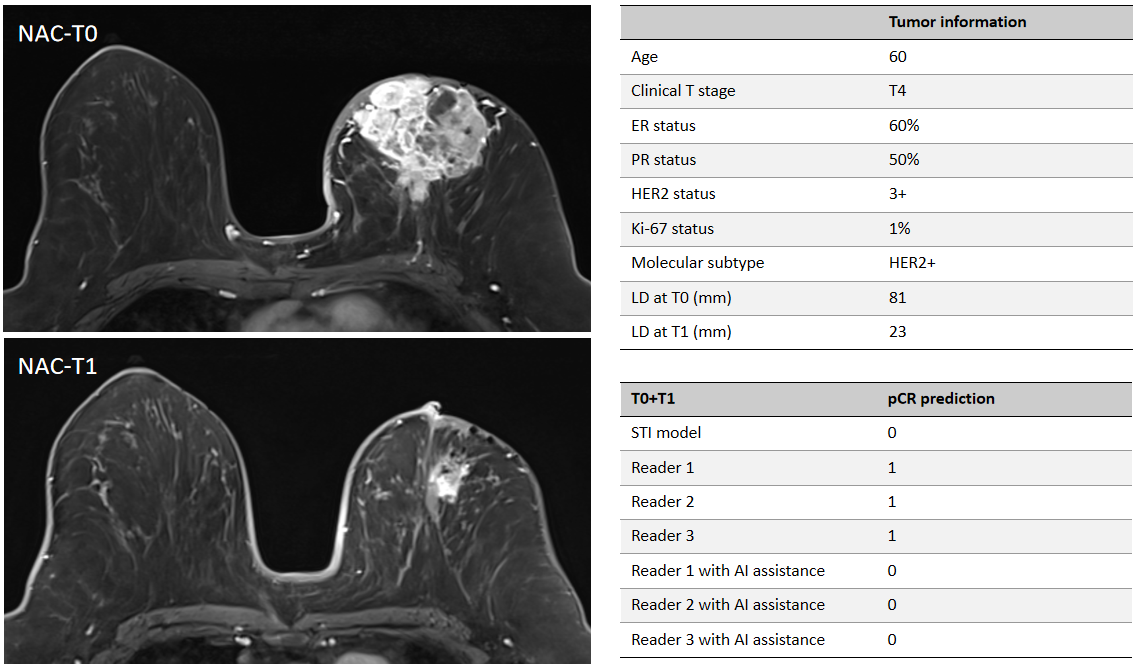


**Fig. S14: Case F.** Another instance of successful human-AI synergy. The STI model correctly identified the patient as non-pCR, and only with the aid of the model did the radiologists update their prediction accordingly.


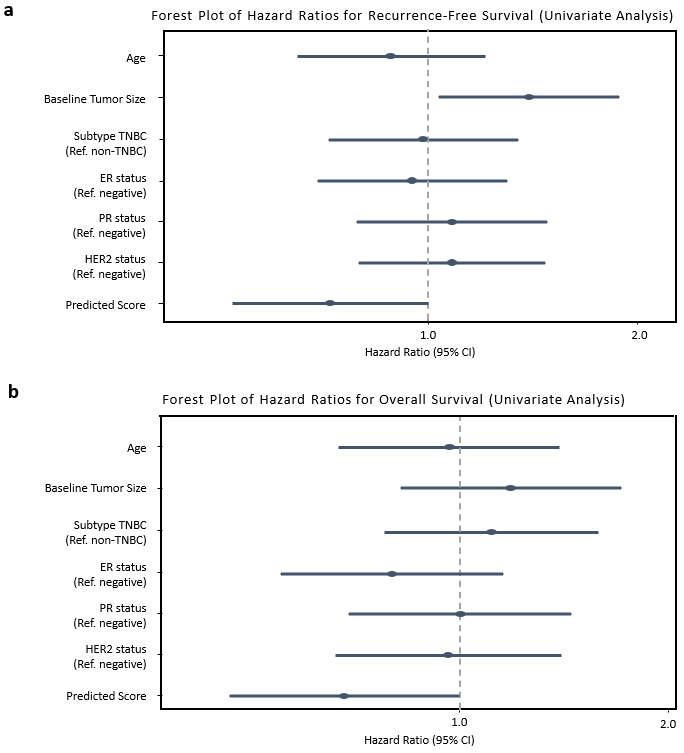


**Fig. S15: Prognostic Significance of the STI Model's NAC Response Predictions.** (a, b) Univariate Cox regression analysis for RFS (a) and OS (b), showing the HR and 95% confidence intervals (CI) for various clinical factors and the STI model's predicted response.

Note: RFS = recurrence-free survival; OS = overall survival; HR= hazard ratio
